# Mucosal TLR5 activation controls healthspan and longevity

**DOI:** 10.1038/s41467-023-44263-2

**Published:** 2024-01-02

**Authors:** Jae Sung Lim, Eun Jae Jeon, Hye Sun Go, Hyung-Jin Kim, Kye Young Kim, Thi Quynh Trang Nguyen, Da Young Lee, Kyu Suk Kim, Federico Pietrocola, Seol Hee Hong, Shee Eun Lee, Kyoung-Shim Kim, Tae-Shin Park, Dong-Hee Choi, Yu-Jin Jeong, Jong-Hwan Park, Hyeon Sik Kim, Jung-Joon Min, Yong Sook Kim, Joon Tae Park, Jae-Ho Cho, Gil-Woo Lee, Ji Hyeon Lee, Hyon E. Choy, Sang Chul Park, Chul-Ho Lee, Joon Haeng Rhee, Manuel Serrano, Kyung A Cho

**Affiliations:** 1 Department of Biochemistry, Chonnam National University Medical School, Hwasun-gun, Jeonnam-do 58128 Republic of Korea; 2 College of Pharmacy and Research Institute of Pharmaceutical Sciences, Chonnam National University, Gwangju, 61186 Republic of Korea; 3 MediSpan, Inc, Bundang-gu, Gyeonggi-do 13486 Republic of Korea; 4 Center for Creative Biomedical Scientists, Chonnam National University Medical School, Hwasun-gun, Jeonnam-do 58128 Republic of Korea; 5 Institute for Research in Biomedicine (IRB Barcelona), Barcelona Institute of Science and Technology (BIST), Catalan Institution for Research and Advanced Studies (ICREA), Barcelona, Spain; 6 Department of Pharmacology and Dental Therapeutics, School of Dentistry, Chonnam National University, Gwangju, 61186 Republic of Korea; 7 Laboratory Animal Resource Center, Korea Research Institute of Bioscience and Biotechnology (KRIBB), Daejeon, 34141 Republic of Korea; 8 Department of Animal Medicine, College of Veterinary Medicine, Chonnam National University, Gwangju, 61186 Republic of Korea; 9 Medical Photonic Research Center, Korea Photonics Technology Institute, Gwangju, 61007 Republic of Korea; 10 Department of Nuclear Medicine, Chonnam National University Hwasun Hospital, Hwasun-gun, Jeonnam-do 58128 Republic of Korea; 11 Biomedical Research Institute, Chonnam National University Hospital, Gwangju, 61469 Republic of Korea; 12 Department of Life Sciences, College of Life Sciences and Bioengineering, Incheon National University, Incheon, 22012 Republic of Korea; 13 Combinatorial Tumor Immunotherapy Medical Research Center, Chonnam National University Medical School, Hwasun-gun, Jeonnam-do 58128 Republic of Korea; 14 Department of Biological Sciences and Bioengineering, Inha University, Incheon, 22212 Republic of Korea; 15 Department of Microbiology, Chonnam National University Medical School, 264 Seoyang-ro, Hwasun-gun, Jeonnam-do 58128 Republic of Korea; 16 Future Life and Society Research Center, Chonnam National University Medical School, Hwasun-gun, Jeonnam-do 58128 Republic of Korea; 17 Altos Labs, Cambridge Institute of Science, Cambridge, UK

**Keywords:** Toll-like receptors, Geriatrics, Protein vaccines

## Abstract

Addressing age-related immunological defects through therapeutic interventions is essential for healthy aging, as the immune system plays a crucial role in controlling infections, malignancies, and in supporting tissue homeostasis and repair. In our study, we show that stimulating toll-like receptor 5 (TLR5) via mucosal delivery of a flagellin-containing fusion protein effectively extends the lifespan and enhances the healthspan of mice of both sexes. This enhancement in healthspan is evidenced by diminished hair loss and ocular lens opacity, increased bone mineral density, improved stem cell activity, delayed thymic involution, heightened cognitive capacity, and the prevention of pulmonary lung fibrosis. Additionally, this fusion protein boosts intestinal mucosal integrity by augmenting the surface expression of TLR5 in a certain subset of dendritic cells and increasing interleukin-22 (IL-22) secretion. In this work, we present observations that underscore the benefits of TLR5-dependent stimulation in the mucosal compartment, suggesting a viable strategy for enhancing longevity and healthspan.

## Abstract (web summary)

Understanding how the immune system’s functionality declines with age is crucial for addressing aging-related health issues. Here, the authors demonstrate that activating TLR5 in the mucosal layer can significantly improve healthspan and longevity, suggesting a novel approach for aging interventions.

## Introduction

Age-associated immunological alterations are multifaced and include enhanced susceptibility to infection and chronic tissue inflammation. Such alterations may contribute to the development of metabolic and cardiovascular diseases, neurodegenerative syndromes, and age-related ailments such as frailty^1–3^. The effects of aging on the immune system appear at various levels, including decreased production of immune cells in the bone marrow and thymus, reduced function of mature lymphocytes in secondary lymphoid tissue, and decreased function of innate immune cells in various tissues^4^. Aging-dependent deterioration of innate immune cells includes the reduction in phagocytic activity, ROS generation, and reduction of pathogen recognition receptors (PRRs) expression, failing to defend against various infections and respond to vaccines^5^. Impaired functions of innate immunity can further exacerbate the flaws in adaptive immunity, for instance, by not providing efficient antigen presentation to T cells^6^. In addition to influenza vaccines, recently, it has been reported that the effectiveness of vaccines against infections such as COVID-19 is reduced in the elderly. Following the BNT162b2 (Pfizer-BioNTech) coronavirus disease 2019 (COVID-19) vaccine, older adults (i.e., ≥80 years old) exhibited decreased IgG antibody titers against an antigen or neutralizing antibody production compared to younger adults (<60 years old)^7^. Since robust innate immune responses are critical for a strong vaccine response, adjuvants for vaccine target PRRs to boost a sufficient inflammatory innate immune response in the elderly.

Toll-like receptors (TLRs) are the major PRRs that induce innate immunity by recognizing pathogen-associated molecular patterns (PAMP) derived from various outer pathogens and are very important in improving vaccines’ efficacy as a target of mucosal vaccine adjuvants^8,9^. However, aging limits the activation of TLRs by agonists following low production of inflammatory cytokines, such as TNFα, interleukin (IL)6, and IL12, due to reduced TLRs expression^10^. In our previous studies, we found that TLR5 expression and signaling were relatively well-preserved in aged mice and older individuals compared to other TLRs^11–13^. As flagellin is a specific TLR5 ligand and an effective mucosal adjuvant that enhances antigen-specific immune responses, we utilized an adjuvant-antigen fusion combination called FP, which consists of flagellin (FlaB, F) fused to pneumococcal surface protein A (PspA, P) and has been previously shown to elicit potent immune responses^14,15^. Vaccination with FP resulted in significant antigen-specific antibody production and provided protection against Streptococcus pneumoniae infection in both young and aged mice^12^. Interestingly, post-vaccination with FP, aged mice exhibited rejuvenating phenotypic changes, leading us to explore the possibility that mucosal immune stimulation through TLR5 signaling could be a strategy to intervene in the aging process and enhance health span and lifespan.

Here, we show that stimulating TLR5 through mucosal delivery of the FP fusion protein positively impacts various aspects of health and physiology in aged mice, suggesting a potential role in extending health span and lifespan.

## Results

### Lifespan Extension by FPNI

FP nasal instillation (FPNI) was initiated in both male and female mice at 650 days of age and continuously administered at two-week intervals. Most functional studies, excluding cognitive function, were conducted after the eighth cycle of FPNI. The lifespan study was carried out until the natural death of the mice (Supplementary Fig. 1). To obtain optimal conditions for substance administration, a mixture of F and P (F + P) or a fusion of F-P (FP) was intranasally administered in eight cycles to 21-month-old mice, and then compared with the PBS-administered control group. We observed that treatment with FP led to a lower frequency of tumors or cirrhosis in the liver (Supplementary Table 1) compared to treatment with a mixture of F and P. Therefore, we selected the FP fusion protein for aging intervention. Observing lifespan first, our findings revealed distinct patterns for both sexes. The Student’s t-test showed a statistically significant difference in mean lifespan for both male and female groups with FP administration (male, *p* value = 0.0416; female, *p* value = 0.0063), while the Wang-Allison test indicated a statistically significant difference in median lifespan for the female group only (*p* value = 0.046). However, no significant difference was observed in either median lifespan (*p* value = 0.5973) or maximal lifespan (*p* value = 0.1906) using the Wang-Allison test (Supplementary Table 2). While the variations in statistical significance between the sexes suggest a complex relationship between the intervention and lifespan, it is important to note that our results collectively demonstrate a multidimensional rejuvenation of aging functions in both groups. This implication that the intervention under investigation may have a broad and substantial impact on improving aging-related outcomes, encompassing various aspects of health span and lifespan, even if there were no significant differences in maximal lifespan between the groups.

### Comprehensive Healthspan Improvements with FPNI

**Fig. 1:**
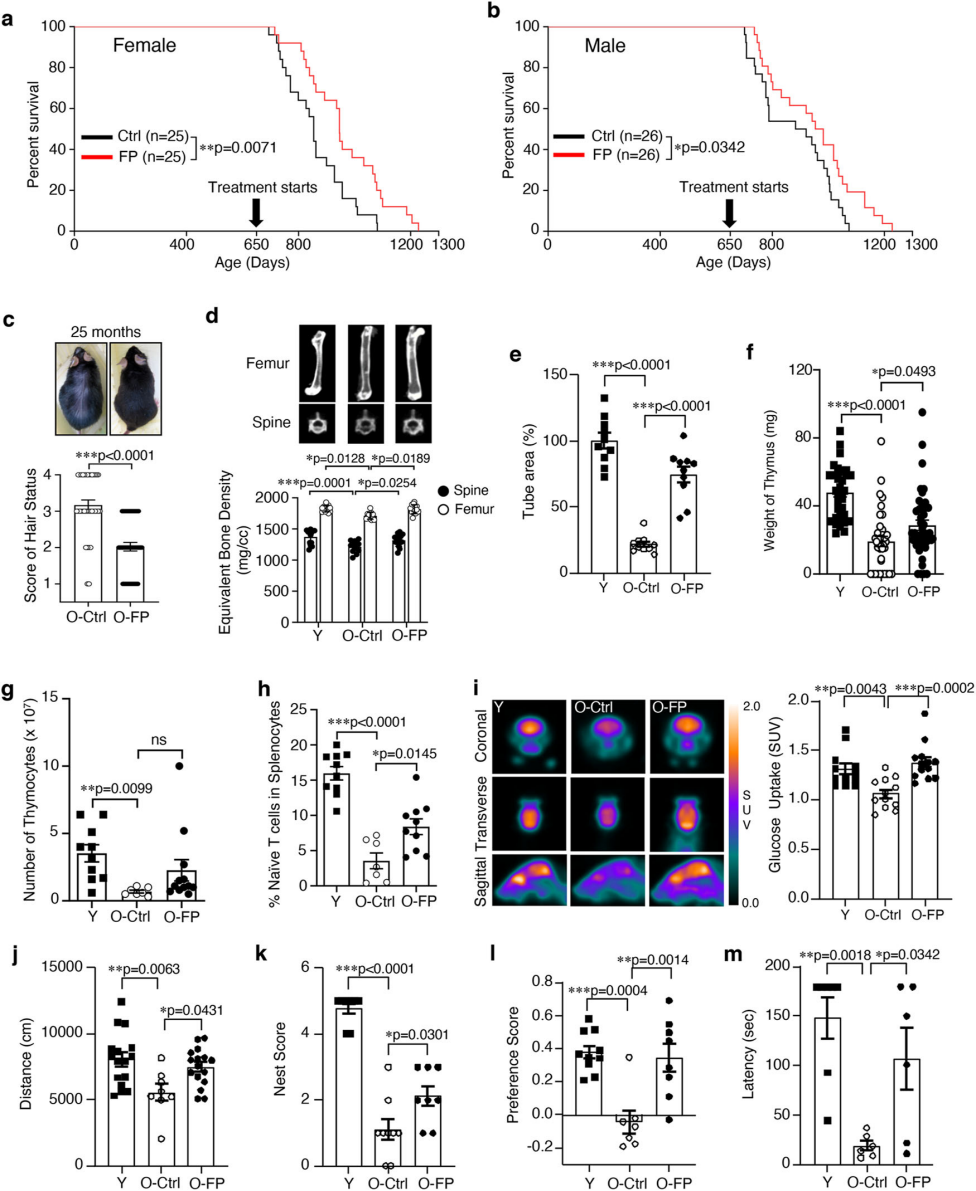
FPNI leads to longevity with a broad range of beneficial health effects. Kaplan-Meier survival graphs for female (**a**, *n* = 25 biologically independent animals per group) and male (**b,** *n* = 26 biologically independent animals per group) mice comparing vehicle control mice to those that received intranasally administered to FP fusion protein (6.5 μg) starting at 650 days of age. After the eight times of FP or vehicle administration, the aged mice were analyzed and compared to those of young mice. The results are displayed below: hair condition **(c**, O-Ctrl: *n* = 36; O-FP: *n* = 43 biologically independent animals), bone mineral density of the spine and femur determined by micro-CT (**d**, Y: *n* = 13; O-Ctrl: *n* = 13; O-FP: *n* = 14 biologically independent animals), total area of tube formation of bone marrow-derived stem cells (**e**, n = 10 biologically independent samples per group), weight of thymus (**f,** Y: *n* = 35 samples; O-Ctrl: *n* = 36 samples; O-FP: *n* = 43 samples), number of thymocytes (**g**, Y: *n* = 10 samples; O-Ctrl: *n* = 7 samples; O-FP: *n* = 12 samples), phenotypic analysis of naïve T cells (CD3^+^CD44^lo^CD62L^hi^) in splenocytes (**h**, Y: *n* = 10 samples; O-Ctrl: *n* = 7 samples; O-FP: *n* = 10 samples), micro-PET images of the mouse brains (left) and quantitative analysis of glucose uptake (right) (**i,** Y: *n* = 12 biologically independent animals; O-Ctrl: *n* = 12 biologically independent animals; O-FP: *n* = 13 biologically independent animals), Cognitive functions were assessed with an open field test (**j**, Y: *n* = 16 biologically independent animals; O-Ctrl: *n* = 8 biologically independent animals; O-FP: *n* = 16 biologically independent animals), the nest score (**k**, Y: *n* = 9 biologically independent animals; O-Ctrl: *n* = 9 biologically independent animals; O-FP: *n* = 8 biologically independent animals), the preference score (**l,** Y: *n* = 10 biologically independent animals; O-Ctrl: *n* = 7 biologically independent animals; O-FP: *n* = 8 biologically independent animals), and the passive avoidance test (**m**, Y: *n* = 7 biologically independent animals; O-Ctrl: *n* = 6 biologically independent animals; O-FP: *n* = 6 biologically independent animals). Error bars represent mean ± SEM. ^*^P < 0.05, ^**^P < 0.01, ^***^P < 0.001 using the Log^-^rank (Mantel-Cox) test (**a** and **b**), the unpaired two-tailed *t* test (**c**), and the one-way ANOVA (**d–m**). ns, not significant; FP, FlaB-PspA fusion proteins; Ctrl, vehicle control group of aged mice; Y, young mice (8 weeks old); SUV, standardized uptake value. Source data are provided as a Source Data file.

To analyze the improvement of healthspan by FPNI, we assessed physical and functional aging phenotypes at various time points following FPNI (Supplementary Fig. 1). We measured hair status, ocular lens opacity, bone mineral density, and bone marrow-derived stem cell activity to gain a comprehensive understanding of the impact of the intervention on aging-related outcomes. After eight cycles, there was a significant protective effect on hair loss and cataracts (Fig. 1c, Supplementary Fig. 2, and Supplementary Table 3) as well as improvement of bone mineral density in both the spine and the femur (Fig. 1d) with a normal spinal curve angle (Supplementary Fig. 3), compared to control aged mice. The lifespan extension and aging phenotype seemed slightly more effective in females but not significantly different. The subsequent functional tests and analyzes were conducted mainly using females. FPNI also led to the recovery of bone marrow-derived stromal cells, as evidenced by tube formation (Fig. 1e). Because FP was originally designed as a mucosal vaccine for older people^12^, we evaluated thymic and splenic cellularity. Both thymus weight and the number of thymocytes were partially restored after FPNI (Fig. 1f, g). The splenic naïve T cell population increased after treatment with FP (Fig. 1h)^16^, and the number of macrophages and dendritic cells decreased to levels similar to those of young mice (Supplementary Fig. 4a, b). Splenomegaly in aged mice was reduced, with lower levels of DNA damage and aging markers such as p53 and p16^INK4a^ compared to the spleen of control aged mice (Supplementary Fig. 5a-c). The degree of health span and lifespan extension by FPNI is comparable to that of rapamycin, when administered starting at 600 days of age^17^. Moreover, to our knowledge, 650 days (when we initiated FPNI) is the latest initiation point for any intervention associated with extended longevity^17,18^. Next, we measured brain glucose absorption using micro-PET and cognitive activities^19,20^. FPNI remarkably improved brain glucose absorption in aged mice to levels similar to those of young mice (Fig. 1i). We also performed open-field tests to investigate the effect of FPNI on spontaneous behavior in aged mice. Aged animals showed a significant decrease in horizontal locomotor activities, nest building, novel object recognition, and passive avoidance tasks compared to young mice (Fig. 1j-m). All of these functions were significantly improved after eight cycles of FPNI.

### FPNI Mitigates Pulmonary Fibrosis

**Fig. 2:**
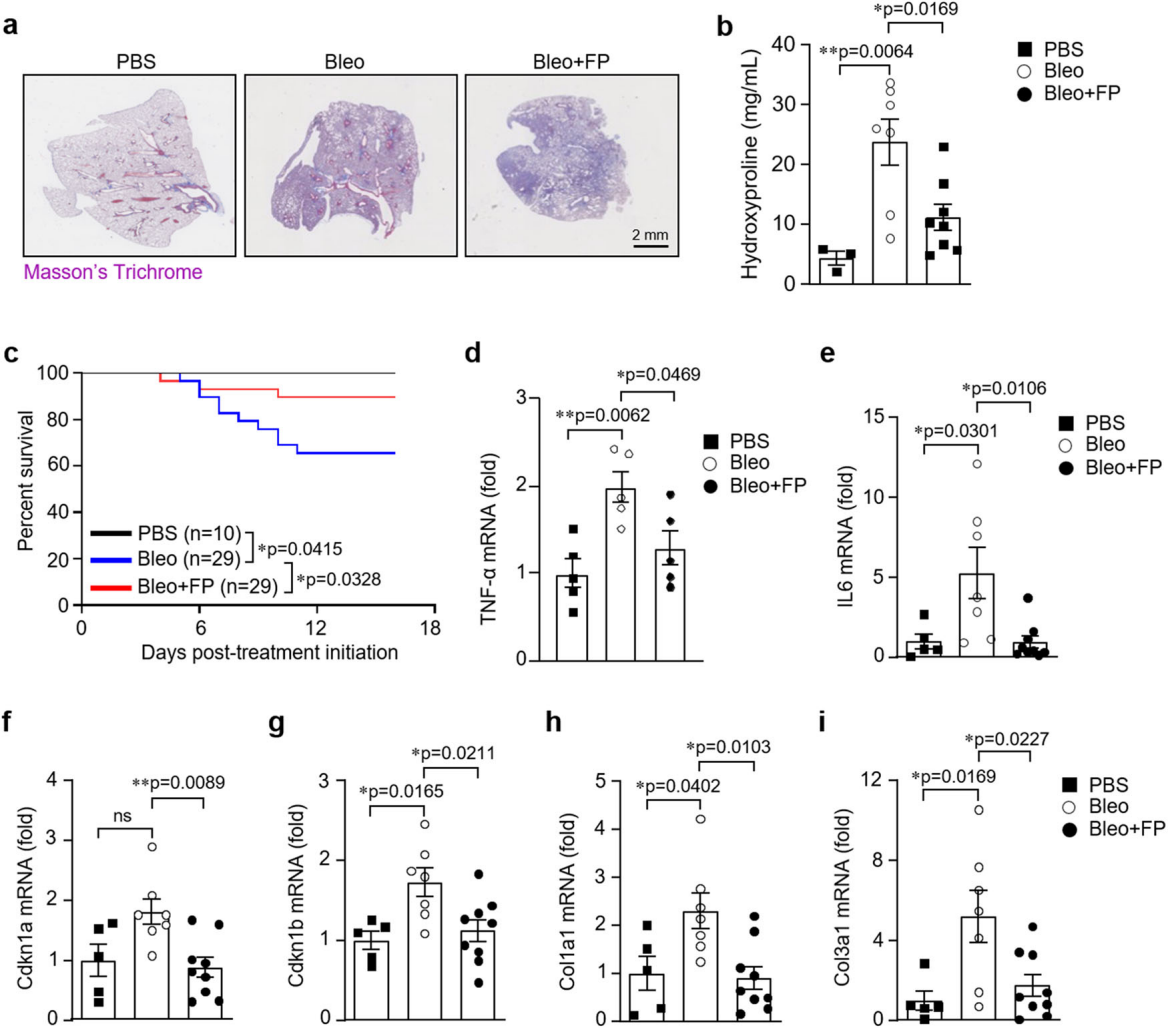
FPNI prevents from the development of Bleomycin-induced lung fibrosis. Pulmonary fibrosis was induced by endotracheal administration of Bleomycin (Bleo) to C57BL/6 J mice as indicated in the corresponding Experimental Procedure section. Phosphate-buffered saline (PBS) was used as a control vehicle. Mice were left untreated or were treated with FP (Bleo+FPNI). The level of fibrosis was assessed at day 16 after Bleo administration. **a** Masson’s Trichrome staining of representative lung sections of mice-treated with PBS (*n* = 3 samples), Bleo (*n* = 7 samples), and Bleo+FPNI (*n* = 8 samples). Scale bar, 2 mm. **b** Quantification of Hydroxyproline content in lungs of mice after treatment with PBS (*n* = 3 samples), Bleo (*n* = 7 samples), and Bleo+FPNI (*n* = 8 samples). (**c**) Survival curves (PBS: *n* = 10 biologically independent animals; Bleo and Bleo+FPNI: *n* = 29 biologically independent animals) of the above-indicated groups of treatment. Relative expression of the mRNA coding for *TNF-α* (**d**), *IL6* (**e**), *Cdkn1a* (**f**), *Cdkn1b* (**g**), *Col1A1* (**h**), and *Col3a1* (**i**) in the lung of mice (PBS: *n* = 5 samples; Bleo: *n* = 7 samples; Bleo+FPNI: *n* = 9 samples) treated as previously indicated. Error bars represent mean ± SEM. ^*^P < 0.05; ^**^P < 0.01 using the one-way ANOVA (**b** and **d–h**) and the Log-Rank (Mantel-Cox) test (**c**). ns, not significant. Source data are provided as a Source Data file.

We wondered whether FPNI could also protect against severe degenerative aging-associated diseases. For this, we decided to investigate the possibility that FPNI could prevent the development of pulmonary fibrosis in mice using the well-established model of intratracheal bleomycin administration. We found that a single FPNI (delivered at day −1 and day 0 prior to bleomycin instillation) could reduce collagen deposition, as measured by Masson’s Trichrome staining (Fig. 2a) and hydroxyproline levels (Fig. 2b) at day 16 post-bleomycin. In line with the anti-fibrotic action, we observed that a single administration of FPNI could significantly extend the survival of bleomycin-treated animals (Fig. 2c). Also, FPNI treatment prevented the upregulation of senescence markers that are well-known to be associated to lung fibrosis, such as *TNF-α* (Fig. 2d), *Il6* (Fig. 2e), *Cdkn1a* (Fig. 2f) and *Cdkn1b* (Fig. 2g) along with that of collagen isoforms *Col1a1* (Fig. 2h) and *Col3a1* (Fig. 2i). All together, these data undercore that FPNI not only delays aging but also offers protection from aging-associated diseases, potentially through its anti-inflammatory effects, underscoring its multifaceted role in promoting healthspan.

### TLR5 activation key to FPNI efficacy

**Fig. 3:**
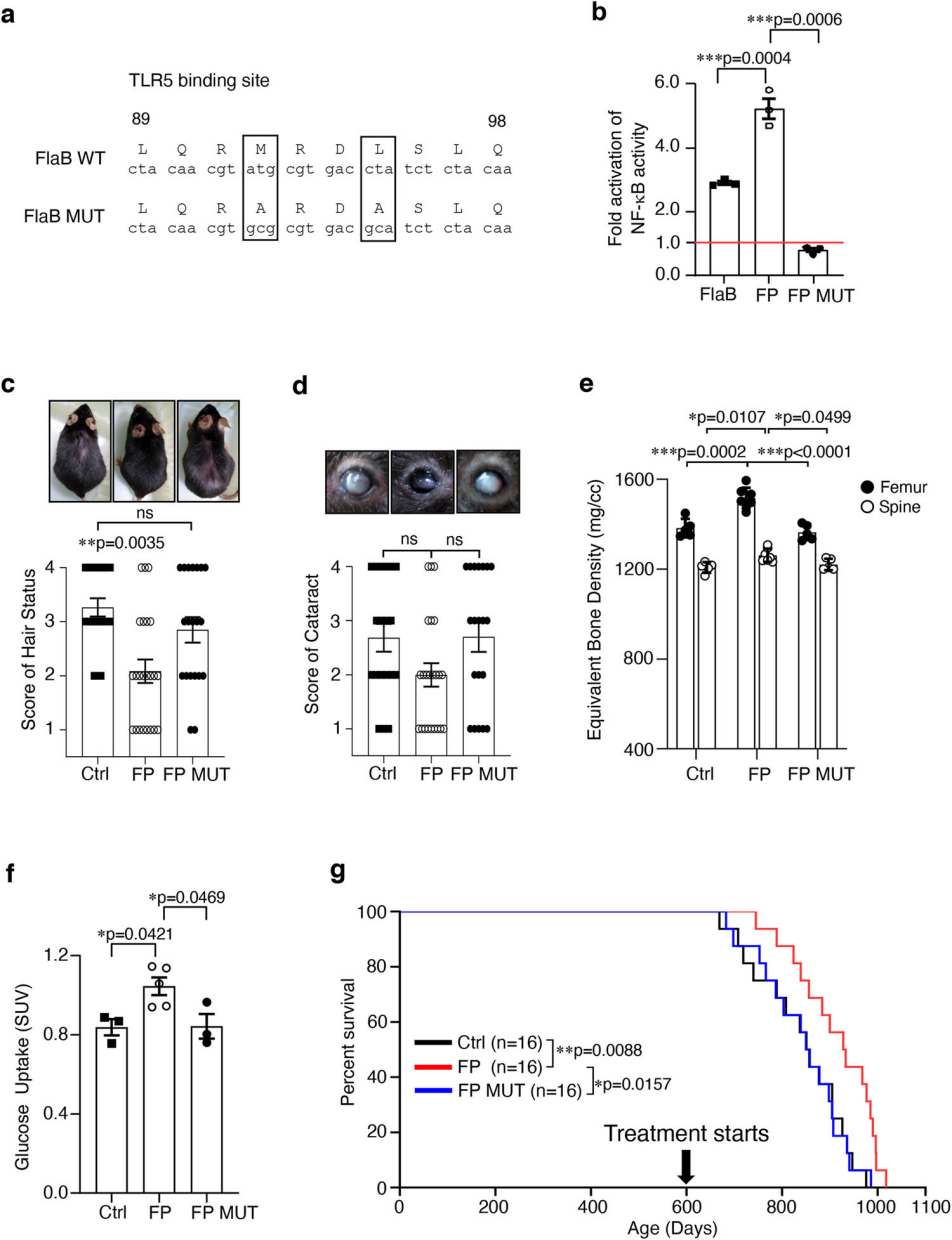
TLR5 plays an essential role in the FPNI-dependent extension of healthspan. Amino acid sequences of the TLR5 binding site (top) and its mutant derivative (bottom) (**a**). The locations of site-directed mutations in the predicted TLR5 binding region are boxed. Activation of NF-κB through TLR5 stimulation with FlaB, FP or FP MUT proteins (**b**, *n* = 3 biologically independent samples per group). After the eight times of FP, FP MUT or vehicle administration their phenotypes were determined; hair status (**c,** O-Ctrl: *n* = 19 biologically independent animals; O-FP: *n* = 23 biologically independent animals; O-FP MUT: *n* = 20 biologically independent animals) and cataracts (**d**, O-Ctrl: *n* = 19 biologically independent animals; O-FP: *n* = 23 biologically independent animals; O-FP MUT: *n* = 20 biologically independent animals), bone mineral density of the spine and femur (**e**, O-Ctrl: *n* = 5 biologically independent animals; O-FP: *n* = 7 biologically independent animals; O-FP MUT: *n* = 5 biologically independent animals), analysis of glucose uptake in the brain (**f,** O-Ctrl: *n* = 3 biologically independent animals; O-FP: *n* = 5 biologically independent animals; O-FP MUT: *n* = 3 biologically independent animals) are displayed in quantitative bar graphs. Survival rates of female mice that received FP or FP MUT intranasally were compared with those of vehicle mice (**g**, *n* = 16 biologically independent animals per group). The survival rates were compared using Kaplan-Meier analysis. Error bars represent mean ± SEM. ^*^P < 0.05, ^**^P < 0.01, ^***^P < 0.001 using the one*-*way ANOVA (**b**–**f**), and the Log-rank (Mantel-Cox) test (**g**). ns, not significant. FlaB, *Vibrio vulnificus* major flagellin; FP MUT, site-directed mutant FP. Source data are provided as a Source Data file.

To understand the mechanisms underlying FP-dependent beneficial health effects in aged mice, we determined the effects of nasal immunization with either PspA or FlaB alone. Individually delivered FlaB or PspA did not have a notable effect on the health span (Supplementary Fig. 6a-d) in aged mice. As FP was designed to target TLR5, we evaluated whether TLR5 is essential for the FPNI-induced improvement in health status in aged mice. Because TLR5 knockout (KO) mice display severe inflammatory problems^21^, we could not generate sufficient numbers of KO mice older than 20 months. Thus, we constructed a site-directed mutation (MUT) at the FlaB domain that disrupts the binding of FlaB to TLR5^22^ and fused the product with PspA (FP MUT) (Fig. 3a and Supplementary Fig. 7a, b). As designed, FP MUT did not bind TLR5 and consequently did not activate NF-κB, a key transcription factor downstream of TLR5 signaling (Fig. 3b) and related immune responses (Supplementary Fig. 7c-e). It was confirmed that PspA alone could not induce the activation of NF-kB by TLR5 (Supplementary Fig. 8), and the TLR5 activity induced by FP was also reduced in FP MUT. However, it was observed that FP could induce higher activity than FlaB alone (Fig. 3b and Supplementary Fig. 8), and it was predicted that FlaB fused with PspA could have a structural advantage in binding with TLR5. Building on our observations of TLR5 activation variations in FP, FlaB, and FP MUT, we aimed to further clarify the role of TLR5 activation by FP. Consequently, we conducted a second independent longevity study, initiated at 650 days of age, and followed by 8 cycles of weekly nasal instillations to delve deeper into this relationship. In particular, we measured survival and aging parameters after nasal administration of FP, FP MUT, or vehicle. Unlike FP, FP MUT failed to improve hair status, cataract, bone mineral density or brain glucose uptake measured after eight cycles of FPNI with two weeks interval (Fig. 3c-f and Supplementary Table 4) and did not extend lifespan (Fig. 3g and Supplementary Table 5). These results suggest that TLR5 is a critical target for FP-induced health span and lifespan improvement in aged mice.

### FPNI’s systemic and intestinal immunomodulatory effects

**Fig. 4:**
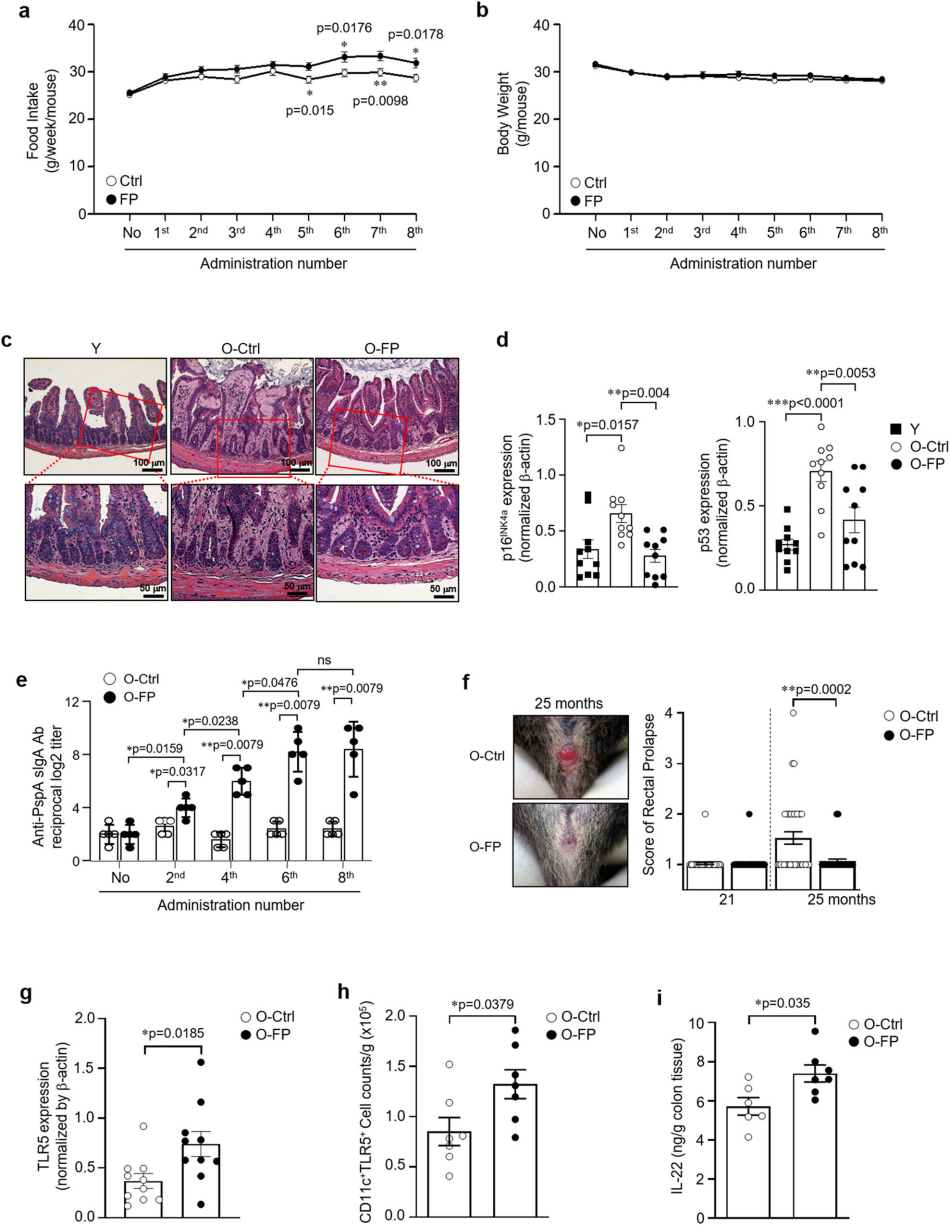
FPNI induces intestinal mucosal immune integrity. During the eight times of FP or vehicle administration, the food intake (**a** Ctrl: *n* = 42; FP: *n* = 50 biologically independent animals) and body weight (**b**, Ctrl: *n* = 45; FP: *n* = 54 biologically independent animals) were measured once a week after each administration in aged mice. The intestine was isolated from young mice and vehicle or FP-administrated aged mice after the eight times of administration. Representative hematoxylin and eosin (H&E)-stained ileum (**c**, *n* = 3 biologically independent samples per group). Upper scale bar, 100 μm; Lower scale bar, 50 μm. The expression of aging marker proteins was determined in the intestine tissues by Western blotting with anti-p16^INK4a^ and anti-p53 antibodies, and the data are represented by the quantitative graphs (**d**, *n* = 10 biologically independent samples per group). Secretory immunoglobulin A (SIgA) levels in the feces were analyzed (**e,** *n* = 5 biologically independent samples per group), rectal prolapse is presented with quantitative bar graphs (**f**, O-Ctrl: *n* = 36; O-FP: *n* = 43 biologically independent animals), TLR5 protein levels in the intestine (**g**, *n* = 10 biologically independent samples per group), TLR5 surface expression in CD11c^+^ LPDCs (**h**, *n* = 7 biologically independent samples per group), IL-22 cytokine levels in tissue explants were analyzed after 5 h of incubation (**i**, O-Ctrl: *n* = 6; O-FP: *n* = 7 biologically independent samples). Error bars represent mean ± SEM. ^*^P < 0.05. ^**^P < 0.01^, ***^P < 0.001 using the two-tailed Student’s *t*-test (**a**), the one-way ANOVA (**d**), and the two-tailed Mann-Whitney *U* test (**e**–**i**). ns, not significant; PspA, surface protein A of *Streptococcus pneumonia*; LPDC, lamina propria dendritic cells. Source data are provided as a Source Data file.

It has been recently reported that TLR5 KO mice display spontaneous colitis, which can induce metabolic syndrome, including hyperlipidemia, hypertension, insulin resistance, and obesity^21,23^. Moreover, intestinal lamina propria dendritic cells (LPDCs) express TLR5 and play a key role in controlling immune homeostasis by producing secretory immunoglobulin A (SIgA) and maintaining tissue structure through interleukin-22 (IL-22)-dependent epithelial regeneration in the intestine^24–29^. Based on this, we wondered if the administration of FP via nasal instillation could reach the intestine and affect its biology. Interestingly, SIgA was continuously increased in feces during FPNI treatment, indicating that nasally-administered FP can reach the intestine, which becomes progressively more responsive to the action of FP (Fig. 4e). FPNI-aged mice showed an increase in food intake but no change in total body weight (Fig. 4a, b) and presented higher voluntary locomotor activity than control mice (see Supplementary Video 1 and Fig. 1j), thus excluding that the cause of lifespan extension was related to a lower caloric intake. Then, we examined whether FPNI-mediated TLR5 activation affected the intestinal epithelium and mucosal immune system. Intestinal villi structures in FPNI-treated aged mice were well preserved, similar to those of young mice, accompanied by the significant reduction of aging markers, such as p53 and p16^INK4a^ after eight cycles of FPNI with two weeks intervals (Fig. 4c, d and Supplementary Fig. 9). Rectal prolapse, a sign of colitis in both TLR5 KO mice^21^ and aged animals, was remarkably prevented by FPNI (Fig. 4f). The total protein amount of intestine and surface expression of TLR5 in a specific subset of LPDCs was elevated by FPNI in aged mice (Fig. 4g, h and Supplementary Fig. 9), and secretion of IL-22 in tissue explants was also significantly increased (Fig. 4i). To further ascertain the role of TLR5, we compared the increase in conventional dendritic cells through FPNI in mesenteric lymph nodes of TLR5 KO mice and observed that while this effect was present in WT (wild type), it was not evident in KO (knockout) mice (Supplementary Fig. 10). These results suggest that FPNI-dependent TLR5 activation led to intestinal mucosal immune system competence and rejuvenated intestinal functions. Furthermore, FPNI also led to an upregulation of TLR5 protein levels as well as a decrease in p16^INK4a^ and p53 protein levels in major organs, such as the lung, liver, and kidney of aged mice (Supplementary Fig. 11a-c). In addition, inflammatory cytokines of IL-6 was remarkably reduced in major organs by FPNI in old mice like those in young mice (Supplementary Fig. 12). These results suggest that TLR5 stimulation via the intranasal route with flagellin has systemic effects that lead to functional restoration in major aged tissues.

## Discussion

In advanced aging, innate immune activation has been viewed as an inducer of chronic inflammation, which promotes different signs of aging and age-related diseases. At the same time, numerous examples indicate that the innate immune system contributes to tissue homeostasis and repair. In this regard, studies in *C. elegans* suggest that innate immunity plays a positive role in longevity by improving pathogen resistance^30,31^.

TLRs are crucial for the innate immune system’s response to threats, primarily by regulating inflammation and activating immune responses. The reduction in TLR activity induced by aging leads to a decreased efficiency in immune responses, potentially lowering the body’s resistance to infections. TLR5 is a remarkably versatile receptor found on both epithelial and immune cells, and its functions are distributed throughout the body. One of its critical roles emerges in the respiratory tract, where TLR5 assumes a pivotal position in initiating protective immune responses, particularly when combating infections like Pseudomonas aeruginosa^32^. It is well known that the reduction in TLR activity induced by aging leads to a decreased efficiency in immune responses, potentially lowering the body’s resistance to infections. However, in our previous study, we discovered that TLR5 expression and signaling were relatively well-preserved in aged mice and older individuals compared to other TLRs^12^. We also demonstrated that TLR5 effectively enhances vaccine efficacy against pneumonia, leading to increased survival rates from pneumococcal infection in old mice. Unlike other TLRs, TLR5 has been reported not only to induce pro-inflammatory signals essential for vaccine efficacy boosting but also to suppress inflammation in lesions, induce tissue regeneration in major disease models, and strengthen the barrier. This unique functionality underscores the diverse applicability and therapeutic potential of TLR5 in addressing age-related health issues and promoting longevity. In several studies, the enhanced liver regeneration facilitated by TLR5-mediated signaling has been highlighted, showcasing its importance in tissue repair and regeneration processes^33^. Additionally, investigations involving TLR5-deficient mice have revealed the absence of basal inflammatory and metabolic defects, yet these mice exhibit impaired responses from CD4 T cells to flagellated pathogens, emphasizing the critical role of TLR5 in immune responses^34^. Another study indicates that even in the absence of the TLR5 gene, environmental factors can significantly influence the gut microbiome profile, thereby affecting metabolic syndrome outcomes^23^. Furthermore, the application of TLR5 agonists has been shown to stimulate hepatocyte proliferation^33^, further illustrating the multifaceted roles of TLR5 in metabolic regulation and immune system modulation^35^. These findings highlight the unique and diversified roles of TLR5, setting it apart from its proinflammatory counterparts and underscoring its potential in age-modulation interventions.

It is well known that TLR5 is essential in maintaining the balance of the intestinal immune system by interacting with E. coli and warding off diseases arising from disruptions in the microbial equilibrium^36^. Furthermore, in the liver, TLR5 makes substantial contributions to the clearance of bacteria and offers significant protection against inflammatory processes^37^. This multifaceted role of TLR5 underscores its significance in diverse body systems and deepens our understanding of its influence on immune responses and overall health. This suggests that TLR5 could serve as a key target for modulating the aging process and associated phenotypes.

Our current data demonstrate that mucosal activation of TLR5 can extend longevity and reduce age-related health defects (Fig. 1). Interestingly, nasal administration of FP in aged mice successfully modulated the intestinal mucosal immune system via TLR5 activation and led to increased production of IgA and IL-22 and retention of young intestinal structure (Fig. 4). Furthermore, FPNI not only increased the expression of TLR5 in various tissues but also inhibited the expression of aging markers, including p16^INK4a^ and p53 (Supplementary Figure 9 and 11). Given the known upregulation of proinflammatory cytokines, IL-6 and TNFa, through TLR5 stimulation by FP-treatment, and their association with the senescence-associated secretory phenotype (SASP), we further investigated the impact of FPNI treatment on these cytokine levels in the bleomycin-induced lung fibrosis model. Our findings revealed a significant attenuation in the upregulation of IL-6 and TNF-α, consistent with the observed reduction in senescence markers. This highlights the multifaceted therapeutic potential of FPNI, suggesting not only its efficacy in mitigating senescence but also its role in modulating inflammation, which is particularly relevant in the context of senescence-driven diseases like lung fibrosis.

Contrary to expectations, administering FlaB alone was not sufficient to ameliorate age-related health defects (Supplementary Figure 6). We clearly demonstrated in vitro that PspA does not directly interact with TLR5, establishing FP as the most potent TLR5 stimulant, followed by FlaB alone (Supplementary Figure 8). Additionally, PspA bound to the FlaB mutant, which is unable to bind to TLR5, did not induce any anti-aging effects (Fig. 3). These results suggest that TLR5 plays an essential role in the anti-aging effects induced by FP, and it is speculated that the enhanced effects of FP compared to FlaB alone may be due to the fusion structure with PspA aiding in the binding to TLR5. A preceding study elucidated that FlaB-PspA fusion proteins inhibit the polymer formation of flagellins, which in turn expose the TLR5-interacting domains more effectively. The FlaB-PspA fusion protein exhibited enhanced potency, more effectively stimulating TLR5 signaling in vitro and more robustly inducing protective immunity in vivo compared to the PspA-FlaB fusion^38^. Additionally, the FlaB-PspA fusion protein is suggested to have a longer half-life, thereby providing more sustained stimulation to immune cells. Previous our research proved that the activity of TLR5 varies depending on the binding order of FlaB and PspA^38^. This study has demonstrated that fusing PspA to the C-terminal of FlaB induces much higher TLR5 activity compared to fusion at the N-terminal. This implies that the C-terminal fusion of PspA is likely more advantageous for the binding structure of FlaB with TLR5. Upon further structural consideration, it can be hypothesized that FlaB, integral to FP, encompasses domains D0, D1, and D2. While the interaction between the N-terminal D1 of FlaB and TLR5 is well-documented, the interaction and structure of the C-terminal D0 remain to be elucidated. Emerging studies indicate that the D0 domain is instrumental in sustaining the dimeric form binding of flagellin and TLR5, which is crucial for TLR5 activation^39,40^. Discoveries of positive residue mutations in the flagellin D0 decreasing TLR5 activity emphasize the significance of charge interactions in this domain. Electrostatic interactions are likely mediating the binding between D0 and TLR5. By binding PspA to the C-terminus of FlaB, FP fosters charge interactions between Flagellin and TLR5, thereby enhancing TLR5 activity. However, further studies would be beneficial to more fully validate this prediction. This comprehensive insight into the mechanism and advantages of FP over other forms suggests its potential as a pivotal agent in TLR5-centric interventions for age modulation. However, while our findings provide a foundation for understanding the role and functionality of FP, it is imperative that further experiments and studies are conducted to validate and expand upon these observations. Additional research will be essential to fully elucidate the underlying mechanisms, optimize the structural and functional characteristics of FP, and explore its therapeutic potential and safety in clinical applications.

This study illuminates the potential of TLR5 as a modulator of aging, necessitating extensive further research. It is imperative to validate the effects observed across diverse strains and elucidate the underlying mechanisms. Moreover, optimizing the structural and functional characteristics of flagellin, such as FP, that stimulate TLR5 is crucial for developing more effective and safe compounds for clinical applications. Understanding the detailed mechanisms by which TLR5 contributes to aging and age-related diseases can further aid in the identification of potential therapeutic targets^41^. Our findings unveil the promising horizon of TLR5-centric interventions, setting the stage for future endeavors aiming at the development of novel therapeutic strategies and a deeper comprehension of aging mechanisms through the lens of immune modulation. The revelation of TLR5’s potential in this study underscores the necessity for continued exploration and refinement, ultimately contributing to the betterment of age-related health outcomes.

## Methods

### Mice

All the mouse procedures were conducted following the guidelines of the Animal Care and Use Committee of Chonnam National University (Approval number: CNU IACUC-H-2019-23). Experimental protocols for lung fibrosis animal model were approved by the Ethical Committee for Animal Experimentation (CEEA) of the Scientific Park of Barcelona (PCB license number CEEA-18-012) and the Government of Catalunya and complied with their ethical regulations. For the lung fibrosis animal experiments, all mice were maintained at the Institute for Research in Biomedicine (IRB) under specific pathogen-free conditions following the recommendations of the Federation of European Laboratory Animal Science Associations (FELASA).

Female and male C57BL/6 J aged mice were generated in-house (by CHL at KRIBB, Korea) or purchased from the Animal Facility of Aging Science at Korea Basic Science Institute (KBSI). Most of the mice received 18 ~ 19 months of aged mice from each site and then used 21 months for each experiment. The detailed scheme of the experimental design is described in Supplementary Fig. 1. All mice were housed in a specific pathogen-free animal facility, maintained on a 12 h light/12 h dark cycle, at a temperature of 22 °C and 45% humidity, with ad libitum access to food and water. Euthanasia procedures were performed under deep anesthesia using isoflurane.

### Purification of FlaB, PspA, and FlaB-PspA recombinant proteins

The recombinant proteins of FlaB, PspA, and fusion FlaB-PspA were prepared as previously described^14,22,38^. Briefly, to amplify each DNA fragment of FlaB and PspA, a DNA fragment containing the open reading frame of FlaB protein from *Vibrio vulnificus* or PspA protein from *Streptococcus pneumonia* were cloned into pTYB12-yielding pCMM250 or pCMM8206 (New England Biolabs, Beverly, MA, USA). To amplify the DNA fragments of fusion FlaB-PspA (FP), DNA fragments of FlaB (F) and PspA (P) were cloned into the pTYB12-yielding pCMM8208 (New England Biolabs). These plasmids were transformed *Escherichia coli* ER2566 strains and induced by isopropyl β-D-1-thiogalctopyranoside (IPTG, 0.4 mM) (New England Biolabs, Beverly, MA, USA) and then purified proteins using chitin column affinity chromatography (New England Biolabs) as manufacturer’s protocol. The purity of recombinant proteins was confirmed by sodium dodecyl sulfate-polyacrylamide gel electrophoresis (SDS-PAGE) and Western blot analysis using in-house produced anti-FlaB and anti-PspA serum raised in rabbits^14,15^. Briefly, 500 μg of purified FlaB or PspA in phosphate-buffered saline (PBS) and mixed with complete Freund’s adjuvant (CFA) (Sigma, CAS9007-81-2) for vaccination. Rabbits were immunized with the purified FlaB or PspA three times with 2-week intervals by muscle injection. Two weeks after final vaccination, the anti-FlaB or anti-PspA serum was collected and tested by western blotting assay for respective antigens.

Lipopolysaccharide (LPS) contamination was removed from the recombinant proteins using the Affinity Pak Detoxi-Gel Endotoxin-Removing gel columns (Pierce Biotechnology Inc., Rockford, IL, USA), and the residual LPS content of the proteins was determined using the gel-clotting Endosafe LAL Kit (Charles River Laboratories Inc., Charleston, SC, USA). The LPS levels in FlaB, PspA, or FP recombinant proteins were less than those in the Food and Drug Administration (FDA) guidelines (<0.15 EU/30 g per mouse). The concentration of purified proteins was determined by Bradford assay.

### Expression vector construction of site-directed mutagenesis of FlaB and purification of site-directed mutant FlaB-PspA (FP MUT) recombinant proteins

Flagellin is the cognate ligand of toll-like receptor (TLR)5^42^. In *P. aeruginosa*, a short stretch of 10 amino acids (amino acids 88–97; LQRIRDLALQ) in the N-terminal region of the flagellin is predicted to be essential for binding to TLR5^43^. Site-specific mutations in the predicted TLR5-binding region of *P. aeruginosa* PAK flagellin reduced their ability to stimulate interleukin-8 release from A549 cells^22^. We identified the conserved TLR5 binding region in *Vibrio vulnificus* FlaB (amino acids 89–98). To abrogate the TLR5 recognition ability of FlaB, we constructed two site-directed mutations in the TLR5-binding region (92 M to A; 95 L to A), as shown in Fig. 3a. Crossover PCR33 generated the mutated FlaB DNA fragment by two sets of primers. The list of primers is as follows: Forward (F)-FlaB-P1: 5ʹ-GAA TTC ATG GCA GTG AAT GTA AAT ACA A-3ʹ, Reverse (R)-FlaB-P2: 5ʹ-TTG TAG AGA TGC GTC ACG CGC ACG TTG TAG G-3ʹ; F-FlaB-P3: 5ʹ-CCT ACA ACG TGC GCG TGA CGC ATC TCT ACA A-3ʹ, and R-FlaB-P4: 5ʹ-CTG CAG TTA GCC TAG TAG ACT TAG CGC-3ʹ. The underlined sequences indicate the restriction enzyme recognition sites. The mutated FlaB DNA fragment was cloned into the plasmid pTYB12 (New England Biolabs). The expected mutation was confirmed by DNA sequencing analysis. The DNA fragment encoding the antigenic PspA polypeptide^38^ was fused to the C-terminal of mutated FlaB and was expressed as FP MUT proteins. The resulting expression plasmid was transformed into *E. coli* ER2566 strains (New England Biolabs) for protein expression. The FP MUT recombinant protein was purified in the same method as mentioned in the above section.

### Mucosal administration and measurement of lifespan

FP (6.5 μg), FP MUT (6.5 μg), and FlaB (4 μg) were reconstituted to a concentration of 16 μl each. The antigens were then administered intranasally to the mice, with 8 μl delivered into the left nasal cavity and 8 μl into the right nasal cavity (Supplementary Movie 2). The intranasal administration was initiated at 650 days of age and given at two-week intervals. Functional changes in the mice were observed after a total of 8 administrations, and cognitive function observations were conducted at the 4th, 7th, and 8th administrations. For lifespan observation, injections were given at two-week intervals, with continuous monitoring throughout the study (Supplementary Fig. 2)^12,38^. Phosphate-buffered saline (PBS) was used as a control. All mice in the aging cohorts were inspected daily. The principal endpoint of life was determined when the mouse was found dead. The Kaplan–Meier survival estimator was used to estimate the survival function from the lifetime data. The median and maximum (age of 90th percentile) lifespan was calculated. The significance of differences in maximum lifespan was assessed using the Wang-Allison method^44^.

### Measurement of food intake and body weight

We measured mice’s food intake and body weight per week during the administration period of the vehicle, FP, or FP MUT in aged mice. To measure food intake in mice, we provided a predetermined amount of food (50 g/week/mouse) in the mouse cage hopper and weighed the remaining food, including any spilled food in the cage, once a week. Body weight was also measured weekly. All external phenotype results were scored by taking pictures of each group with marking numbers only, and information about each group was not disclosed to the observer.

### Analysis of the phenotypes score in mice

After 8 times of administration, the physical conditions of mice, such as hair status, rectal prolapse, and cataracts, were inspected and assessed on the basis of the frailty index (FI)^45,46^ and applied to the scoring system that was divided into grades from 1 (good) to 4 (severe) depending on each phenotype condition.

#### For the evaluation of hair status

We modified previously reported hair loss scoring systems to fit our model^46,47^. Briefly, the hair status was evaluated and scored depending on the hair condition, such as ‘shiny’, ‘fur’, ‘piloerection’, and an advanced degree of hair loss on the back. A score of 1 denotes a shiny, well-kept, bright hair coat and no detectable hair loss. A score of 2 indicates slight shedding, revealing some gray hair, and an unkempt coat with few small patches or less than 10% hair loss on the back. A score of 3 was assigned to mice with gray hair, a dull coat, and mild piloerection with several small patches or 20%-50% hair loss on the back. Finally, a score of 4 was given to the mice with bristled, clumped, and dull fur with piloerection and larger patches or generalized hair loss (not patchy) exceeding 50% of back hair loss.

#### For the assessment of rectal prolapse severity

We modified previously reported rectal prolapse scoring systems to our needs^48,49^. Briefly, the severity of rectal prolapse was scored by a progressive degree of protrusion from the anus. A score of 1 indicates no detectable rectal prolapse. A score of 2 indicates mild rectal prolapse. A score of 3 indicates moderate prolapse, and a score of 4 represents severe rectal prolapse.

#### For the assessment of cataracts

Cataracts were scored by a progressive degree of opacification in the posterior lens using a previously described opacification grading system^50,51^. Briefly, a score of 1 indicates no opacity sign in the lens. A score of 2 indicates a slight degree of opacification or a randomly shaped lesion (amorphous pattern) in the lens. A score of 3 indicates a diffuse opacification or a more significant lesion of amorphous pattern in the entire lens. A score of 4 indicates an extensive thick opacification (hyper-mature cataract) in the entire lens.

### Measurement of bone mineral density

The right femur and spine were isolated from the adherent soft tissues and fixed in 10% neutral formalin. The right femur and lumbar vertebrae (L3) were scanned for bone mineral density (BMD) by micro-computed tomography (CT)^52,53^. We drew a global region of interest (ROI) and defined the values using 30 slices approximately 0.3 mm from the distal femur and L3 in the spine. Then, the BMD was calculated by the Hounsfield Unit (HU) value in the global ROI. For accurate analysis of BMD, HU values of the micro-CT images were converted to equivalent bone density (mg/cc) by the CT calibration method provided by Siemens. The values of BMD were estimated as the mean converted equivalent bone density within the global ROI.

### Analysis of kyphosis

For analysis of kyphosis, the spine was dissected and fixed in 10% neutral formalin. The angle of spinal curvature was measured using the analysis of Cobb’s angle as defined by the lines drawn to the seventh cervical vertebra (C7), maximal curvature, and the fifth lumbar (L5) vertebral endplates^54,55^. The angle of spinal curvature was calculated using ImageJ software (National Institutes of Health, Bethesda, MD, USA).

### Micro-CT image processing

Micro-CT images for measurement of kyphosis and BMD were obtained using a micro-CT scanner (Inveon, Siemens Medical Solutions, Malvern, PA, USA), which uses multiple axial X-rays of the animal to generate cross-sectional information and a 3-dimensional reconstruction of the animal or parts of the animal. The micro-CT system has a variable focus X-ray source, which provides resolution of up to 15 μm and a 125 mm detector capable of scanning an entire mouse in a single scan. Analysis of kyphosis and BMD measurement in micro-CT images was performed with PMOD software version 3.310 (PMOD Technologies Ltd., Zurich, Switzerland).

### Analysis of tube formation

An in vitro angiogenesis assay was performed as previously described^56^. Briefly, tube formation was determined using an in vitro angiogenesis kit (Merck Millipore, Darmstadt, Germany). The isolated bone marrow-derived mesenchymal stem cells were seeded onto matrix gel-coated 96-well plates (BD Bioscience, San Diego, CA, USA) and cultured in serum-free media. Three replicated wells were set up for each group. Tube formation was examined using a phase-contrast microscope (Nikon, Japan), and the network’s total length was measured using Image-Pro Plus 6.0 (Media Cybernetics, Silver Spring, MD, USA). The angiogenic activity was quantified by measuring tube length. The total tube length in four fields per well was averaged.

### Isolation of splenocytes and thymocytes

The spleen and thymus were dissected and cut into small pieces. To isolate splenocytes or thymocytes from individual mice, tissues were dissociated in RPMI 1640 medium supplemented with 10% FBS, 1% P/S, 1% L-glutamine, and 0.1% β-mercaptoethanol. After harvesting single-cell suspensions through Cell Strainers (60 μm, BD Biosciences), the cells were washed with flow cytometry (FACS) staining buffer (eBioscience, San Diego, CA, USA) and incubated for 2 min in ACK lysing buffer (Gibco), followed by centrifugation and washing with PBS, and resuspension in FACS buffer for FACS analysis.

### Flow cytometry analysis

Single-cell suspensions were prepared from the spleen and thymus. For cell surface staining of various markers in splenocytes and thymocytes or lamina propria cells, the cells were blocked with Fc receptor (FcR) blocking reagents in FACS buffer and stained with different combinations of fluorochrome-conjugated antibodies in FACS staining buffer for 20 min at room temperature (RT) in the dark. All of the following antibodies for FACS were purchased from BD Biosciences or Novus Biologicals: CD3-FITC, CD44-PE-Cy7, CD62L-APC, TLR5-FITC (Novus Biologicals, Centennial CO, USA). FACS data were acquired using a FACS AriaII instrument (BD Biosciences) and analyzed with FlowJo software (Tree star, Ashland, OR, USA).

### Analysis of glucose uptake and micro-PET image processing

Positron emission tomography (PET) images were obtained using a high-resolution small animal PET scanner (Inveon preclinical PET scanner, Siemens Medical Solutions) with 20 × 20 lutetium oxyorthosilicate (LSO) crystals, each measuring 1.5 × 1.5 × 10 mm^3^. The system comprised 64 detector blocks arranged in four contiguous rings, with a detector ring diameter of 16.1 cm, a transaxial field of view (FOV) of 10 cm, and an axial FOV of 12.7 cm. Acquired data were sorted into 3D sinograms or directly into 2D sinograms. 3D sinograms were then rebinned by the Fourier algorithm (FORE). The acquired 3D sinograms were reconstructed in 3D using the 3D ordered-subset expectation maximization (OSEM3D) algorithms for which data were reconstructed using 16 subsets and any of four iterations. The mice anesthetized by applying isoflurane (3%) mixed with oxygen. [^18^F] FDG (7.4 MBq/200 µl) was injected into the tail vein. After 30 min, the mice were placed in the microPET scanner for image acquisition and data were collected for 10 min. The collected data were reconstructed by OSEM3D method.

### Behavior test

Behavior tests were performed between 2 and 12 days after the fourth, seventh, or eighth intranasal administration with FP.

#### Open field test

The open field test has been described in a previous study^57^. Briefly, the locomotor activity was measured in an open field of a white Plexiglas chamber (45 × 45 × 40 cm). Mice were habituated in the test room for 30 min. Each mouse was placed individually at the center of the open field, and the horizontal locomotion was recorded for 30 min. The test was performed using a computerized video-tracking system (SMART; Panlab S.I., Barcelona, Spain).

#### Nesting building

The mice were housed in single cages containing sawdust for 5 days. On the first day of testing, one piece of cotton (5 × 5 cm, Nestlets, Ancare, Bellmore, NY, USA) was introduced in the home cage to permit nesting. The presence and quality of nesting were rated 1 day later on a 5-point scale ranging from 1 to 5 as follows: 1 = nestlet not noticeably touched (>90% intact), 2 = nestlet partially torn up (50%-90% remaining intact), 3 = mostly shredded but often no identifiable nest site, 4 = an identifiable but flat nest, and 5 = a (near) perfect nest. Immediately afterward, the mice were group-housed as before^58^.

#### Object recognition test

Novel object recognition is a validated and widely used test for assessing recognition memory^59,60^. Mice were individually placed in a 40 × 20 × 20 cm^3^ testing chamber for 10 min with two identical objects (familiar, acquisition session). Then, the mice were returned to home cages and 1 day later placed back in the testing chamber in the presence of one of the original objects and one novel object (novel, recognition session) for 10 min. The original objects consisted of cylindrical wooden blocks measuring 10 cm high × 2 cm diameter. The novel object consisted of a rectangular wooden block measuring 10 × 2.5 × 2 cm. The acquisition and recognition sessions were video recorded, and an observer who was blinded to drug treatment scored the time spent exploring the objects. The chambers and objects were cleaned with ethanol between trials. Exploration was defined as sniffing and touching the object with the nose and/or forepaws. Sitting on the object was not considered exploratory behavior. In preliminary studies, naive mice exhibited no significant preference for the cylindrical or rectangular block. The time of exploring both objects was calculated. A preference score was calculated for each animal and expressed by the ratio of (time spent exploring the novel object-time spent exploring the familiar object)/(total time spent with both objects) on day 2^61^.

#### Passive avoidance test

The test apparatus consisted of a light and a dark chamber (17 × 22 × 20 cm), each equipped with a shock-grid floor and a door between the two chambers. During the first day of testing, each mouse was placed in the light chamber and left to habituate to the apparatus for 5 min, while allowing it to explore the light and dark rooms. On the second day, the mice were placed in the light chamber. After 20 sec, the door was opened, and the mice were allowed to enter into the dark chamber such that all four paws were inside. When the mice entered the dark room, the door was closed, and one successive electric foot-shock (0.1 mA, 2 sec) was delivered through the grid floor. After training, the mice were then returned to their home cages. After 7 days, the mice were individually replaced in the light chamber, and the latency to enter the dark chamber was measured, which was recorded as the posttest latency^62^. The cut-off time for the entry into the dark chamber was 180 sec.

### Histological analysis and immunohistochemistry

The spleen tissues were dissected and fixed in 10% neutral formalin and embedded in paraffin. Histological analysis was performed on paraffin-embedded sections stained with hematoxylin and eosin (H&E) using the conventional method. For immunohistochemistry, we performed general methods. Briefly, paraffin tissue sections were deparaffinized and rehydrated with xylene and serial dilutions of ethanol. Then, tissue rehydration was followed by antigen retrieval through boiling in 1 mM citric acid buffer (pH 6.0). Tissue sections were incubated in 3% H_2_O_2_ and blocked with 10% normal serum (Vector Labs, Burlingame, CA, USA) and incubated with γ-H2AX primary antibody (Abcam, Cambridge, MA, USA) overnight at 4 °C. Secondary antibody incubations were carried out using Dako LSAB2 System-HRP (Dako, Santa Clara, CA, USA). Tissue sections were visualized with a DAB system (Vector Labs) and counterstaining was performed by H&E. Images were captured using an Aperio ImageScope instrument (Leica Biosystems Inc., Buffalo Grove, IL, USA).

### Western blot analysis

Tissues were homogenized in RIPA buffer and sonicated. Total proteins were collected after centrifugation. Protein samples were separated using SDS-PAGE and transferred onto polyvinylidene fluoride membranes. The membranes were incubated with anti-TLR5 (Abcam, ab13876, 1:1000), anti-p16^INK4a^ (Invitrogen, MA5-17142, 1:1000), anti-p53 (Santa Cruz Biotechnology, sc-126, 1:1000), and anti-β-actin (Santa Cruz Biotechnology, sc-47778, 1:1000) antibodies overnight in a cold room. The membranes were then incubated with peroxidase-conjugated anti-rabbit and anti-mouse secondary antibodies (Santa Cruz Biotechnology, sc-2357, 1:3000; and Cell Signaling Technology, 7076, 1:3000) for 1 hr at RT and then visualized using an enhanced chemiluminescence detection kit (Amersham ECL Kit; GE Healthcare, Buckinghamshire, UK). Protein expression was analyzed with ImageJ software (National Institutes of Health).

### Pulmonary fibrosis animal experiments

To induce pulmonary fibrosis, 8-weeks-old male C57BL/6 J mice were anesthetized by intraperitoneal injection with ketamine (75 mg/kg) and medetomidine (1 mg/kg). The animals were placed on a Tilting WorkStand for rodents (EMC Hallowell) and intubated intratracheally with a 24GA catheter (BD Biosciences). Bleomycin (Sigma, St.Louis, MO, USA) was intratracheally inoculated at 1.5 U/kg of body weight. FP (6.5 μg) was administered via intranasal route 1 day before, on the same day and 1 week after Bleomycin inoculation. Lung samples were embedded in paraffin and serial sections of the lung were stained with Masson’s Trichrome to assess the presence of collagen.

### Hydroxyproline assay

After euthanasia, lungs were snap-frozen in liquid nitrogen. Lungs were then resuspended in PBS and homogenates were prepared by means of a tissue homogenizer (Fats Prep-24 5 G) (MP Biomedical, Irvine, CA, USA). Total collagen levels were quantified using a Hydroxyproline Assay Kit (Chondrex, Redmond, WA, USA) as indicated by the manufacturer.

### Quantitative-real time PCR

Total RNA was extracted using TRIzol™ Reagent (Thermo Fisher Scientific, Waltham, MA, USA) as indicated by the manufacturer. After quantification in a Nanodrop, RNA was retrotranscribed into cDNA by means of iScript cDNA synthesis kit (BioRad, Hercules, CA, USA). Quantitative Real Time PCR was performed using a Quant Studio 6 Flex System (Thermo Fisher Scientific). Relative RNA expression was normalized using the housekeeping gene Gapdh. The list of qPCR primers is as follows: F-IL6: 5’-GTT CTC TGG AAA TCG TGG A-3’, R-IL6: 5’-GGT ACT CCA GAA GAC CAG AGG A-3’; F-Cdkn1a: 5’-GCA GAT CCA CAG CGA TAT-3’, R-Cdkn1a: 5’-GGA ACA GGT CGG ACA TCA-3’; F-Cdkne1b: 5’-TCA AAC GTG AGA GTG TCT AAC G-3’, R-Cdkn1b: 5’-CCG GGC CGA AGA GAT TTC TG-3’; F-Cola1: 5’-TAG GCC ATT GTG TAT GCA GC-3’, R-Col1a1: 5’-ACA TGT TCA GCT TTG TGG ACC-3’; F-Col3a1: 5’-TAG GAC TGA CCA AGG TGG CT-3’, R-Col3a1: 5’-GGA ACC TGG TTT CTT CTC ACC-3’; F-IL-1β: 5’-GCT GTG GAG AAG CTG TGG CATCT GCT-3’, R-IL-1β: 5’-GTC CGA CAG CAC GAG GCT TT-3’; F-TNF-α: 5’-AGA ACT CCA GGC GGT GCC TA-3’, R-TNF-α: 5’-AGT GTG AGG GTC TGG GCC AT-3’; F-CXCL2: 5’-GGC TAC AGG GGC TGT TGT GG-3’, R-CXCL2: 5’-ACC GCC CTT GAG AGT GGC TA-3’; F-Gapdh: 5’-TTG ATG GCA ACA ATC TCC AC-3’, R-Gapdh: 5’-CGT CCC GTA GAC AAA ATG GT-3’

### NF-κB luciferase reporter assay

TLR5-stimulated NF-κB activity was estimated as previously described^38^. Briefly, HEK293T cells (ATCC, CRL-3216) were transfected with p3XFlag-hTLR5^63^ and pNF-κB-Luc plasmid^64^ using Effectene (QIAGEN, Hilden, Germany). Luciferase activity was normalized to lacZ expression levels using the control expression plasmid pCMV-β-Gal (Clontech, Palo Alto, CA, USA). At 24 hr after the transfection, cells were incubated with FlaB (100 ng), FP (100 ng) or site-directed mutant FP (FP MUT) (100 ng) proteins at the same molar ratio for 24 hr. Cells were lysed with a lysis buffer (Promega, Madison, WI, USA), and luciferase activity was measured by a luminometer (MicroLumatPlus LB 96 V; Berthold, Wilbad, Germany).

### Determination of TLR5-dependent NF-κB activation

The TLR5-dependent NFκB stimulating activity of the recombinant proteins was measured using HEK-Blue^TM^ hTLR5 cells (InvivoGen, hκb-htlr-5) and HEK-Blue^TM^ Detection (InvivoGen, hb-det3) assay systems, following the manufacturer’s instruction. EC50 was calculated using quadruplicate OD 650 nm values for each protein concentration over a wide range of protein concentrations (0.015 nM to 100 nM).

### Analysis of PspA-specific SIgA or IgG antibody response

To determine PspA-specific secretory IgA (SIgA) production, we collected the fecal samples every two weeks during the eight times of administration with FP or vehicle. To determine PspA-specific IgG production, the serum was collected from the administered mice at two weeks after the third administration. PspA-specific SIgA or IgG response was determined by enzyme-linked immunosorbent assay (ELISA). The ELISA was performed as previously described^33^. Briefly, feces were lysed with blocking buffer containing 0.05% Tween-20, 1 mM ethylenediaminetetraacetic acid (EDTA), and 0.5% bovine serum albumin (BSA) and centrifuged for 30 min at 13,000 rpm, and then the supernatants were collected. For separation of serum, blood was collected from the heart and incubated for 20 min at RT. The supernatant was harvested after centrifugation at 600 x g for 20 min at 4 °C. ELISA plates (Corning Laboratories) were coated with PspA protein (1 µg/mL) in PBS for 24 hr at 4 °C. The plates were washed with PBS-T (0.05% Tween20 in PBS) and blocked with blocking buffer at RT for 2 hr. The serially diluted feces samples of sera samples were added into the plates and incubated for 2 hr at RT and then washed with PBS-T. The HRP-conjugated anti-mouse IgG (SouthernBiotech) or anti-rabbit IgA (SouthernBiotech) antibodies was used as the secondary antibody. SIgA or IgG was detected by adding TMB (3, 3’, 5, 5’-tetramethylbenzidine) substrate solution (BD Bioscience). The absorbance was read on a microplate reader (Molecular Devices Corp., Menlo, CA, USA) at 450 nm. The titers were represented as the reciprocal log2 value of the dilution that yielded an optical density of 0.1 at 450 nm.

### Isolation of cells from MLNs and lamina propria

For isolation of cells from MLNs, the abdominal cavity was carefully opened to locate the mesenteric lymph nodes. Subsequently, the mesenteric lymph nodes were aseptically removed and placed in a sterile container containing cold phosphate-buffered saline (PBS). Cell suspensions from the mesenteric lymph nodes were generated through mechanical disruption. For the evaluation of conventional dendritic cells (cDCs), enzymatic digestion was carried out using collagenase VIII (400 U/ml) for 25 minutes at 37 °C. Following enzymatic digestion, the cell suspension was transferred to a centrifuge tube. Centrifugation was conducted at a specific speed and duration at 37 °C to pellet the cells. The supernatant was carefully removed, and the cell pellet was resuspended in an appropriate cell culture medium for subsequent analysis.

Cells from small intestine lamina propria were collected and isolated according to the instructions of the Lamina Propria Dissociation Kit instructions (Miltenyi Biotec, Bergisch Gladbach, Germany) following the manufacturer’s protocol. Briefly, the small intestine of each mouse containing the same area was collected and weighed. Peyer’s patches and fat were removed, and the tissue was flushed with cold PBS and cut into 0.5-cm pieces. Epithelium was dissociated by incubation for 40 min at 37 °C with gentle shaking in Hank’s Balanced Salt Solution (HBSS) without Ca^2+^/Mg^2+^ and containing 5% FBS, 5 mM EDTA and 1 mM dithiothreitol (DTT). The remaining tissue was incubated with the components of the Lamina Propria Dissociation Kit in HBSS containing Ca^2+^/Mg^2+^ and 5% FBS for 30 min at 37 °C with gentle shaking. After incubation, the tissue was dissociated using a gentle MACS Dissociator (Miltenyi Biotec), releasing the lamina propria cells. Then, the cells were collected after passing through a 70-μm cell strainer (BD Bioscience) for FACS analysis and washed in PBS supplemented with 0.5% BSA.

### Analysis of IL-22 cytokine levels from colon explants

Interleukin (IL)−22 cytokine levels in the cultured media of colon explants (CEs) were determined by ELISA as previously described^65,66^. Briefly, 3- to 4-cm colonic samples were weighed and washed in RPMI 1640 medium containing 1% P/S. The CEs were cultured for 5 hr in complete RPMI 1640 at 37 °C in a 5% CO_2_ incubator. The levels of IL-22 cytokines in culture supernatants were then measured using a sandwich Mouse IL-22 DuoSet ELISA kit (R&D systems, Minneapolis, MN, USA).

### Statistical analysis

Statistical analysis was performed using Prism 8 software (GraphPad, Inc., San Diego, CA, USA) and R4. 2. 2. software. Differences between the experimental groups were analyzed by the chi-squared test (χ^2^ test), the two-tailed Student’s *t*-test, the one-way ANOVA, and the Mann-Whitney *U* test. Survival rates were analyzed by the Kaplan-Meier survival curves and the statistical significance was determined by the two-tailed Student’s *t*-test, the one-way ANOVA, and Wang-Allison test for Supplementary Table 2 and 5. The data are represented as the mean ± SEM, except survival data, and were considered statistically significant when *P* values were <0.05.

### Reporting summary

Further information on research design is available in the Nature Portfolio Reporting Summary linked to this article.

### Supplementary information

Supplementary Information

Description of Additional Supplementary Files

Supplementary Video 1

Supplementary Video 2

Reporting Summary

### Source data

Source data

## Data Availability

All relevant data are available within the article and its supplementary information/Source Data. Source data are provided with this paper.
