# Supplementary material for: Mucosal TLR5 activation controls healthspan and longevity
Source: Nat Commun. 2024 Jan 2;15:46. doi: 10.1038/s41467-023-44263-2 (PMC10761998; doi:10.1038/s41467-023-44263-2)
Supplement: Supplementary file 1 — Supplementary Information [file 41467_2023_44263_MOESM1_ESM.pdf]

Supplementary Information

Mucosal TLR5 activation controls healthspan and longevity

Lim JS. *et al.*

This file includes:

Supplementary Figures 1 to 16

Supplementary Tables 1 to 5

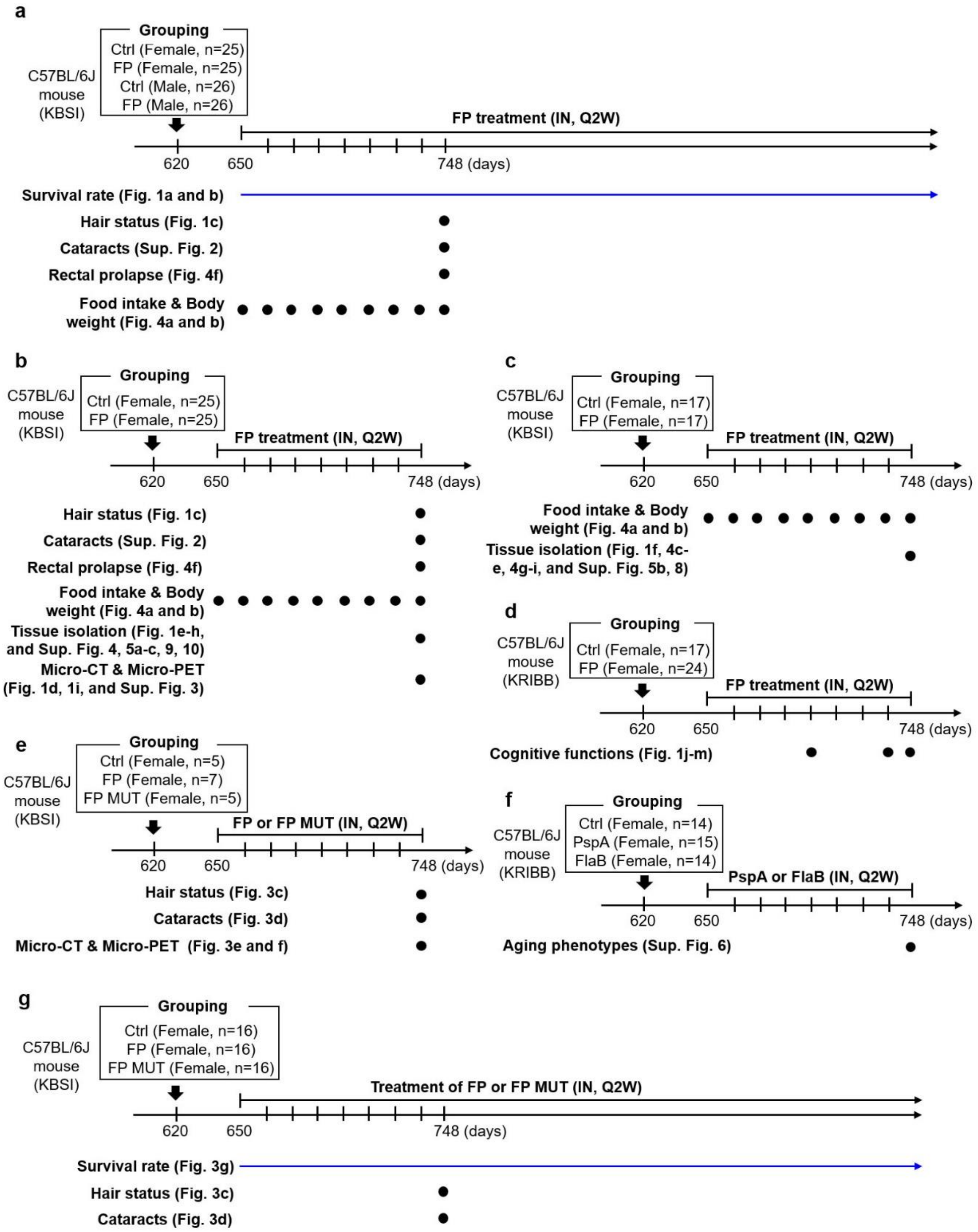
a
Grouping
Ctrl (Female, n=25)
FP (Female, n=25)
Ctrl (Male, n=26)
FP (Male, n=26)
C57BL/6J mouse (KBSI)
FP treatment (IN, Q2W)
620
650
748 (days)
Survival rate (Fig. 1a and b)
Hair status (Fig. 1c)
Cataracts (Sup. Fig. 2)
Rectal prolapse (Fig. 4f)
Food intake & Body weight (Fig. 4a and b)
b
Grouping
Ctrl (Female, n=25)
FP (Female, n=25)
C57BL/6J mouse (KBSI)
FP treatment (IN, Q2W)
620
650
748 (days)
Hair status (Fig. 1c)
Cataracts (Sup. Fig. 2)
Rectal prolapse (Fig. 4f)
Food intake & Body weight (Fig. 4a and b)
Tissue isolation (Fig. 1e-h, and Sup. Fig. 4, 5a-c, 9, 10)
Micro-CT & Micro-PET (Fig. 1d, 1i, and Sup. Fig. 3)
c
Grouping
Ctrl (Female, n=17)
FP (Female, n=17)
C57BL/6J mouse (KBSI)
FP treatment (IN, Q2W)
620
650
748 (days)
Food intake & Body weight (Fig. 4a and b)
Tissue isolation (Fig. 1f, 4c-e, 4g-i, and Sup. Fig. 5b, 8)
d
Grouping
Ctrl (Female, n=17)
FP (Female, n=24)
C57BL/6J mouse (KRIBB)
FP treatment (IN, Q2W)
620
650
748 (days)
Cognitive functions (Fig. 1j-m)
e
Grouping
Ctrl (Female, n=5)
FP (Female, n=7)
FP MUT (Female, n=5)
C57BL/6J mouse (KBSI)
FP or FP MUT (IN, Q2W)
620
650
748 (days)
Hair status (Fig. 3c)
Cataracts (Fig. 3d)
Micro-CT & Micro-PET (Fig. 3e and f)
f
Grouping
Ctrl (Female, n=14)
PspA (Female, n=15)
FlaB (Female, n=14)
C57BL/6J mouse (KRIBB)
PspA or FlaB (IN, Q2W)
620
650
748 (days)
Aging phenotypes (Sup. Fig. 6)
g
Grouping
Ctrl (Female, n=16)
FP (Female, n=16)
FP MUT (Female, n=16)
C57BL/6J mouse (KBSI)
Treatment of FP or FP MUT (IN, Q2W)
620
650
748 (days)
Survival rate (Fig. 3g)
Hair status (Fig. 3c)
Cataracts (Fig. 3d)

Supplementary Figure 1. Experimental design scheme. This scheme outlines a series of experiments conducted using C57BL/6J mice, which were randomly assigned to various treatment groups at 620 days of age. From day 650 onwards, treatments were administered intranasally at bi-weekly intervals, utilizing the following formulations: phosphate-buffered saline (PBS) as a vehicle control, PBS containing Pneumococcal surface protein A (PspA, 2.5 μg), flagellin B (FlaB, 4 μg) from Vibrio vulnificus, a fusion protein of FlaB and PspA (FP, 6.5 μg), a site-directed mutant of FP (FP MUT, 6.5 μg), and a combination of FlaB (4 μg) with PspA (2.5 μg). (a, b, e-g) Health parameters were assessed after four months, which corresponds to eight treatment sessions. (a, g) The survival of the mice was monitored until natural death. (a-c) Food intake and body weight metrics were recorded bi-weekly. (d) Cognitive functions were evaluated after the fourth, seventh, and eighth treatments. For the longevity experiment, a single cohort of mice was observed for lifespan and visible aging symptoms such as hair loss, cataracts, and prolapse. FP, FlaB-PspA fusion proteins; FP MUT, site-directed mutant FP; PspA, surface protein A of *Streptococcus pneumonia*, FlaB, *Vibrio vulnificus* major flagellin. The red arrow indicates FP treatment events, the blue arrow marks the measurement of survival rates, and the black dots signify experimental milestones.

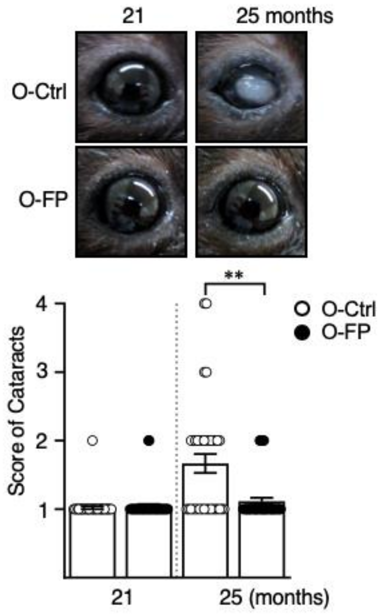

Supplementary Figure 2. Cataract incidences by FPNI in aged mice. After treatment with FP or vehicle in old mice, symptoms were photographed and scored according to the standard score (described in the methods) and displayed in a graph. (n=36 animals/O-Ctrl; n=43 animals/O-FP). Error bars represent mean ± SEM. **P=0.0019 using the χ2 test. FP, FlaB-PspA fusion proteins; Ctrl, vehicle control group of aged mice. Source data are provided as a Source Data file.

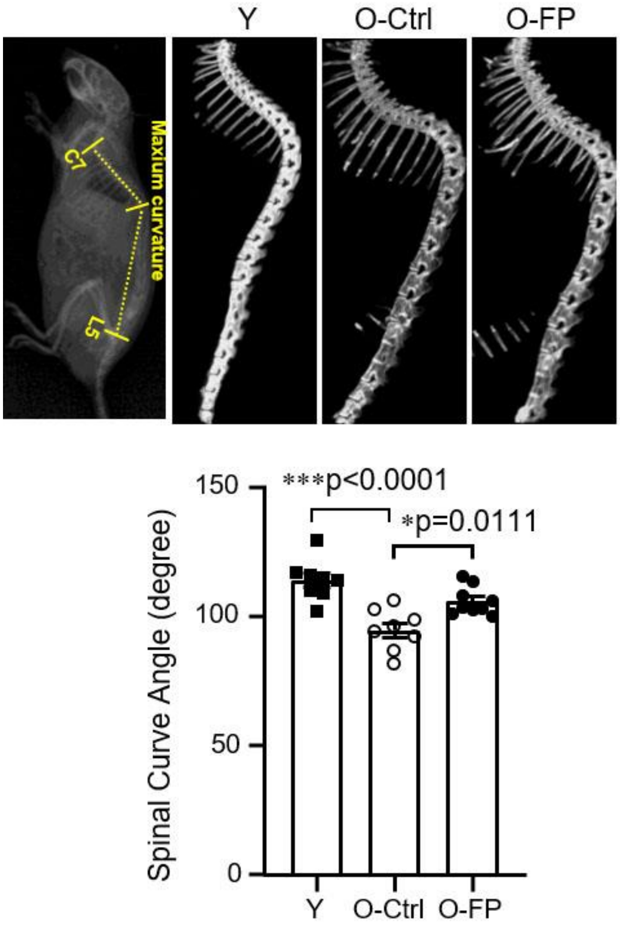

Supplementary Figure 3. FPNI prevents kyphosis in aged mice. The angle of curvature was measured using the analysis of Cobb angle as defined by the dotted lines and represented in a graph (n=8 animals/Y; n=8 animals/O-Ctrl; n=9 animals/O-FP). Error bars represent mean ± SEM. $^{*}P < 0.05$, $^{***}P < 0.001$ using the one-way ANOVA with Tukey's test for multiple comparisons. Y; young mice. C7, seventh cervical vertebra; L5, fifth lumbar vertebra. Source data are provided as a Source Data file.

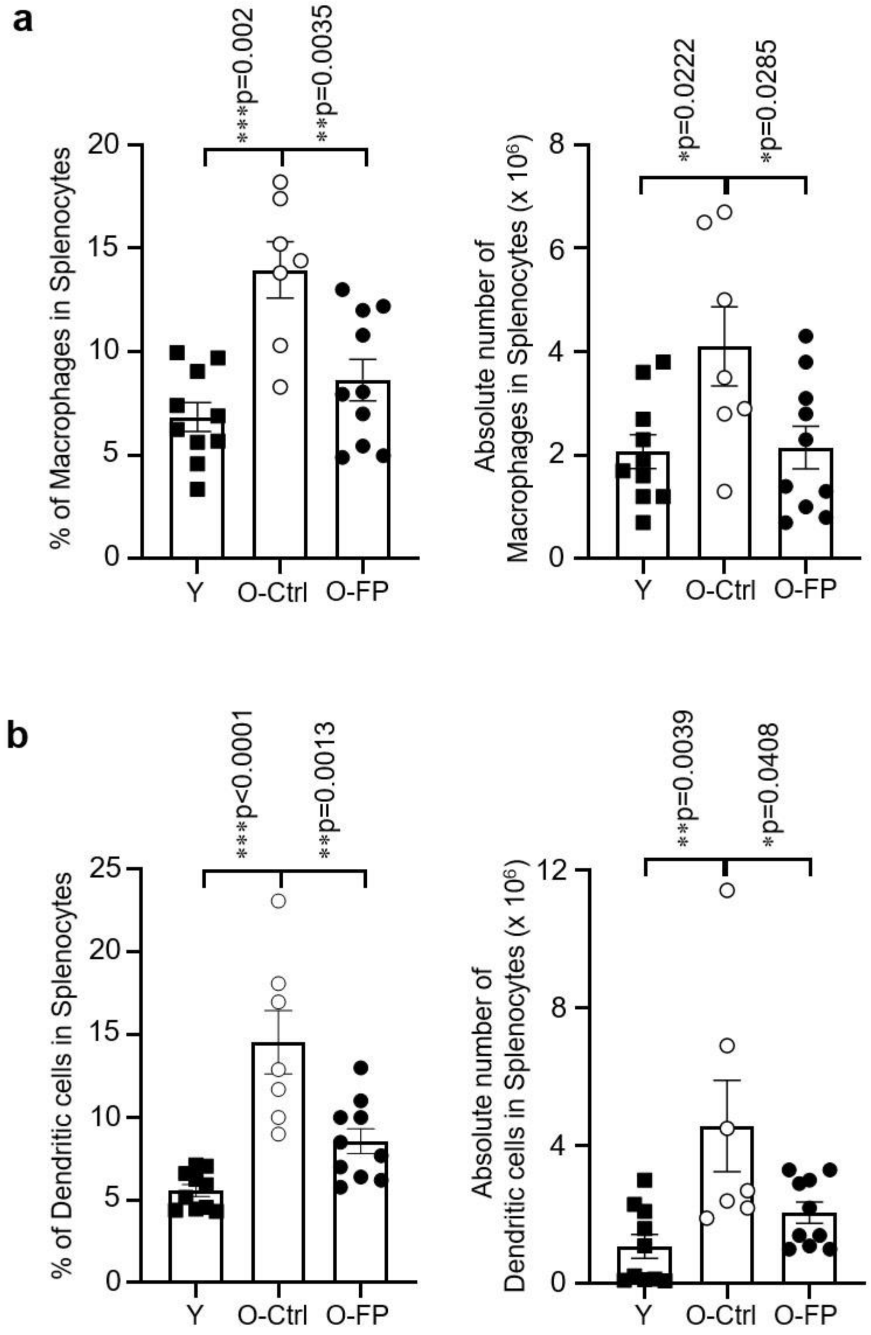

Supplementary Figure 4. Splenic immune cells were compared in young, vehicle or FP-administered old mice. The numbers of (a) macrophages ($CD11b^{+}MHCII^{+}F4/80^{+}$) and (b) dendritic cells ($CD11c^{+}MHCII^{+}$) were analyzed by flow cytometry. The data are displayed by plot graphs (n=10 samples/Y; n=7 samples/O-Ctrl; n=10 samples/O-FP). Error bars represent mean ± SEM. $^{*}P < 0.05$, $^{**}P < 0.01$, $^{***}P < 0.001$ using the one-way ANOVA with Tukey's test for multiple comparisons. Source data are provided as a Source Data file.

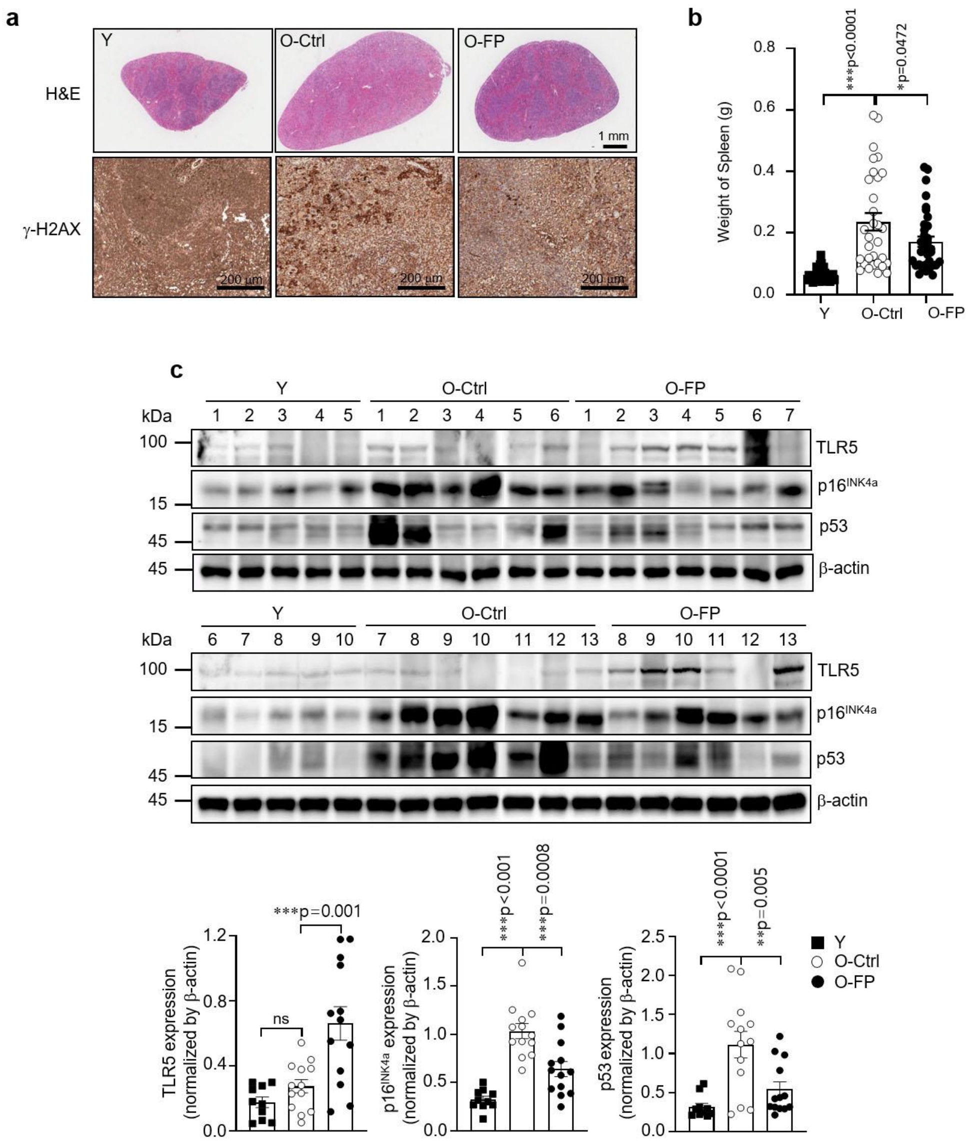

Supplementary Figure 5. Effect of FPNI in the spleens of aged mice. (a) Representative hematoxylin and eosin (H&E)-stained spleens (top; Scale bar, 1 mm) and immunohisto chemical staining (bottom; Scale bar, 200 μm) show DNA damage in spleens with anti-γ-H2AX antibody (n=3 biological independent repeats). (b) The weight of spleen (n=30 samples/Y; n=31 samples/O-Ctrl; n=34 samples/O-FP). (c) The expression of aging marker proteins was determined in the spleen (n=10 samples/Y, n=13 samples/O-Ctrl; n=13 samples/O-FP) by Western blotting with anti-TLR5, anti-p16^{INK4a}, and anti-p53 antibodies. β-actin was used as the loading control. The data are represented by quantitative graphs. Error bars represent mean ± SEM. $^{**}P < 0.01$, $^{***}P < 0.001$ using the one-way ANOVA with Tukey's test for multiple comparisons (b). ns, not significant. Source data are provided as a Source Data file.

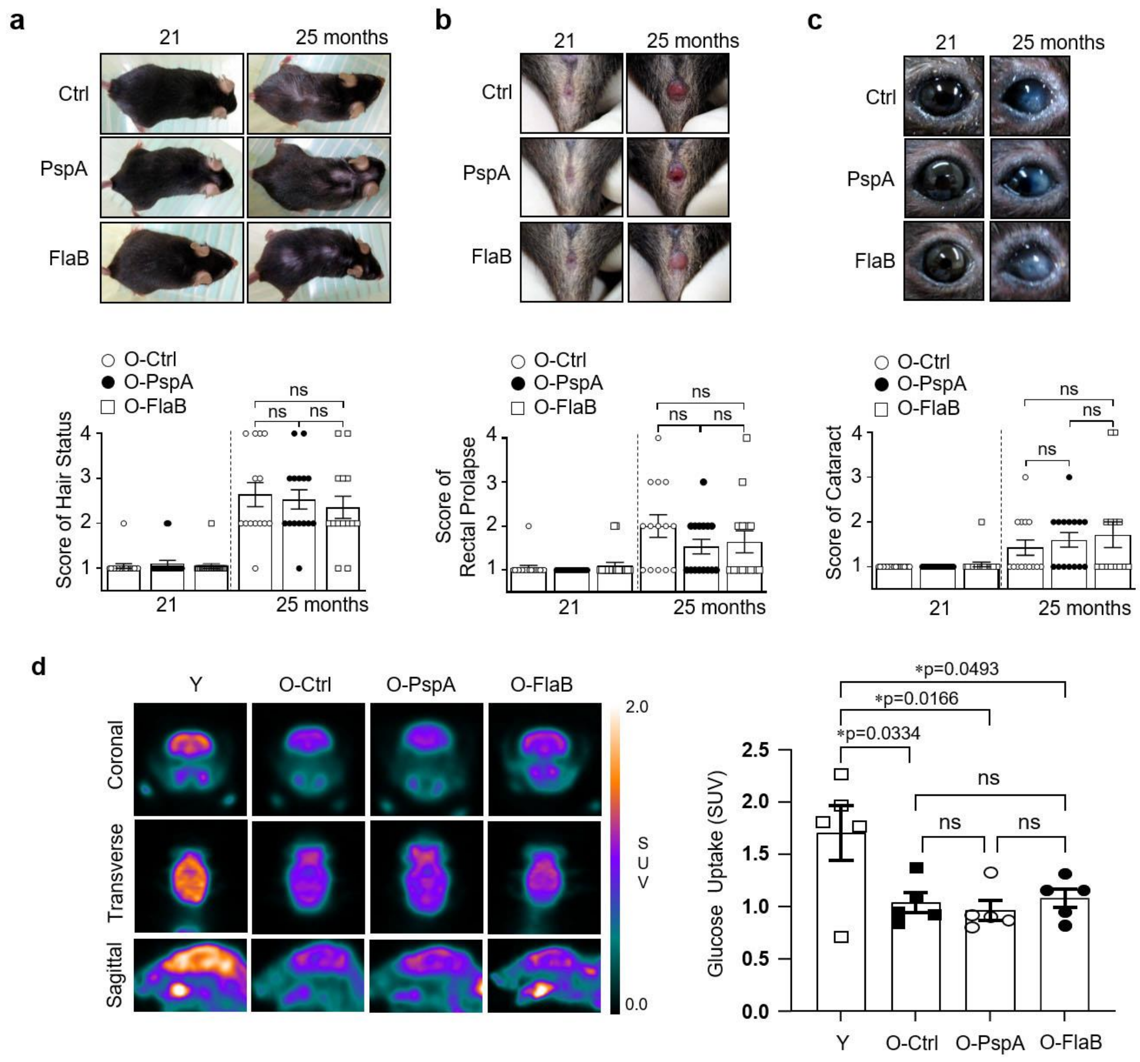

Supplementary Figure 6. Aging phenotypes by intranasal administration with PspA or FlaB in old mice. After treatment of PspA (2. 5 µg) or FlaB (4 µg) via intranasal route, hair condition (a), rectal prolapse (b), and cataract (c) (n=14 animals/O-Ctrl; n=15 animals/O-PspA; n=14 animals/O-FlaB) were analyzed. (d) Representative [^{18}F] FDG micro-PET images in mouse brains (left) and quantitative analysis of glucose uptake (right) (n=5 biological independent repeats). Error bars represent mean ± SEM. *P<0.05 using the one-way ANOVA with Tukey's test for multiple comparisons. ns, not significant. PspA, surface protein A of *Streptococcus pneumoniae*. FlaB, *Vibrio vulnificus* major flagellin. Source data are provided as a Source Data file.

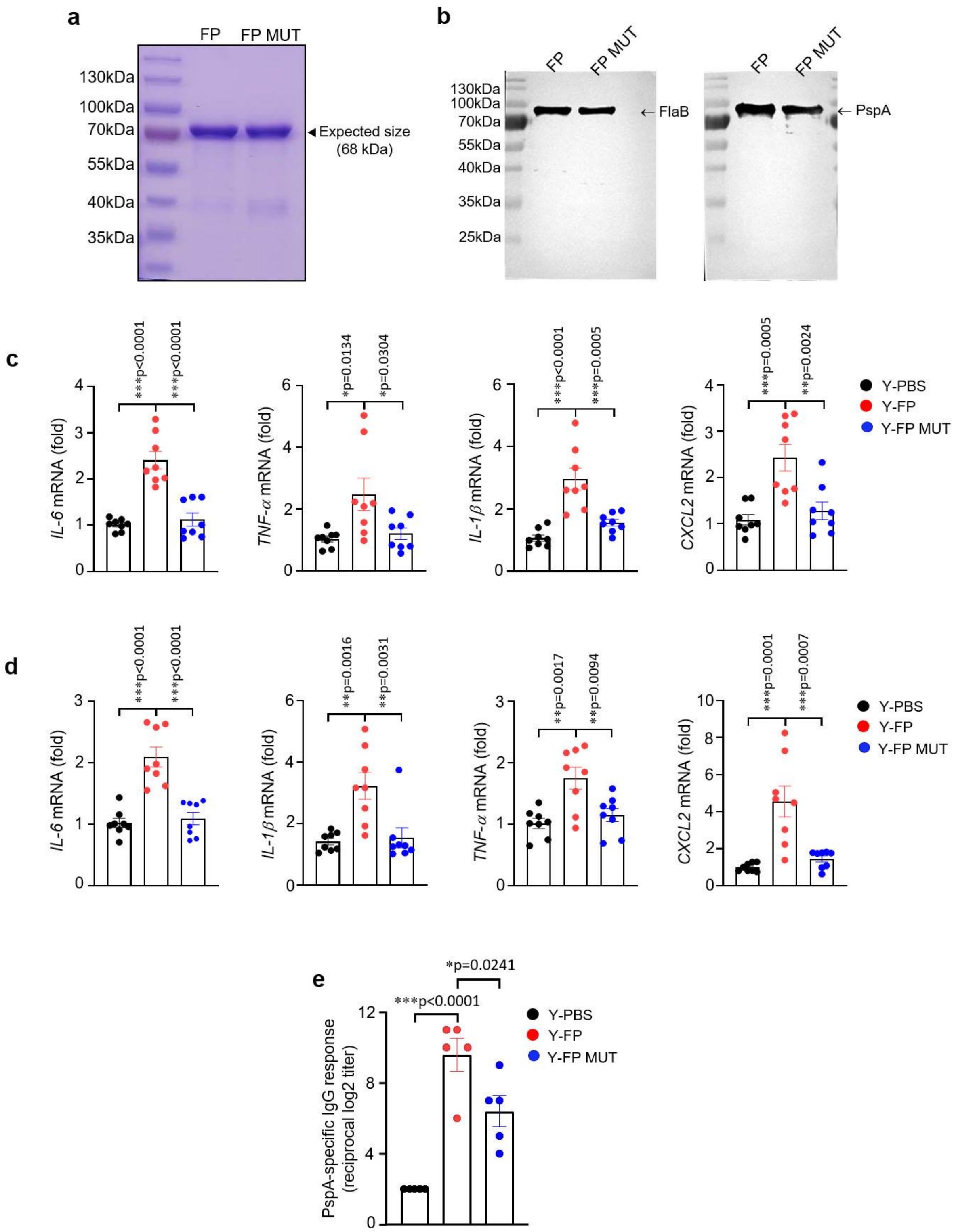

Supplementary Figure 7. Innate immune response and antibody response induced by administration of FP recombinant proteins. In order to elucidate the efficacy of FP and FP MUT, we compared the expression of proteins, PspA-specific antibody production, and expression of various inflammatory cytokines using newly purified FP and FP MUT. The

purity of FP- and FP MUT-recombinant proteins was confirmed by SDS-PAGE (a) and the Western blot analysis using in-house produced rabbit anti-PspA or anti-FlaB serum (b). Next, to compare the innate immune responses and antibody responses in vivo with FP- and FP MUT-recombinant proteins, we administered FP- and FP MUT-recombinant proteins to young mice via intranasal three times at 2-week intervals. At 6 hr after three rounds of recombinant proteins, we checked the innate immune responses through tissue culture by extracting cervical lymph node (cLN) and spleen in young mice administered with recombinant proteins. The innate immune responses were activated with the increase in the expression of pro-inflammatory cytokines such as *IL-6*, *TNF-α*, *IL-1β*, and *CXCL2* in cLN (c) and spleen (d) of FP-administrated mice but not FP MUT (n=8 biological independent repeats). PspA-specific IgG responses were significantly increased in the serum of FP-administrated mice, while it was decreased in FP MUT-administrated mice (e, n=5 biological independent repeats). These results suggest that FP recombinant proteins are well induced the innate immune responses or antibody responses in vivo, while FP MUT recombinant proteins are not effective in their responses. The data represented by the quantitative graphs. Error bars represent mean ± SEM. $^{*}P<0.05$, $^{**}P < 0.01$, $^{***}P < 0.001$ using the one-way ANOVA with Tukey's test for multiple comparisons. Source data are provided as a Source Data file.

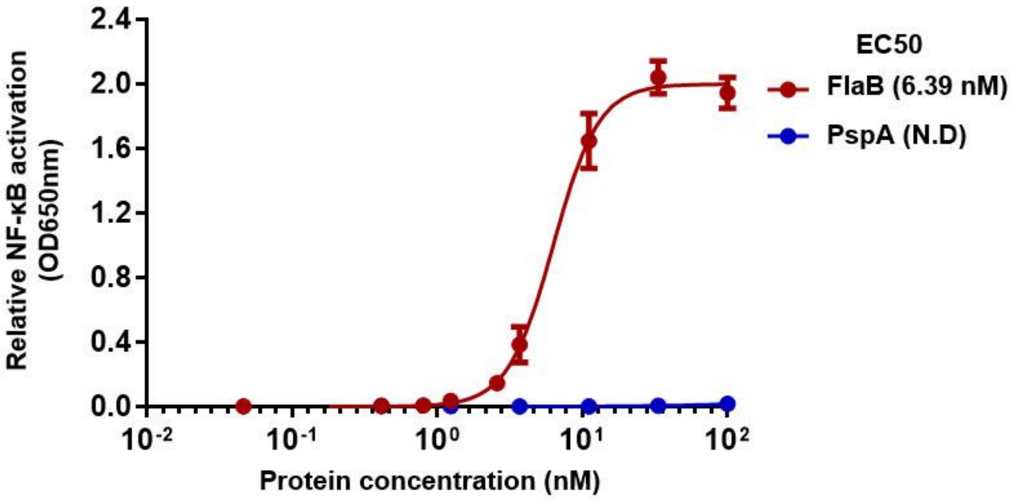

Supplementary Figure 8. Effects of FlaB and PspA on TLR5 activity. Determination of TLR5-dependent NF-κB stimulating activity by FlaB and PspA. The relative NF-κB activities were analyzed by using HEK-Blue™ hTLR5 cells and HEK-Blue™ detection assay systems (n=4 biological independent repeats). EC50 were calculated using triplicate OD 650 nm values for each protein concentration over a wide range of protein concentrations (0.015 nM to 100 nM). The same molar ratio of proteins was used, and PBS was used as a negative control. Source data are provided as a Source Data file.

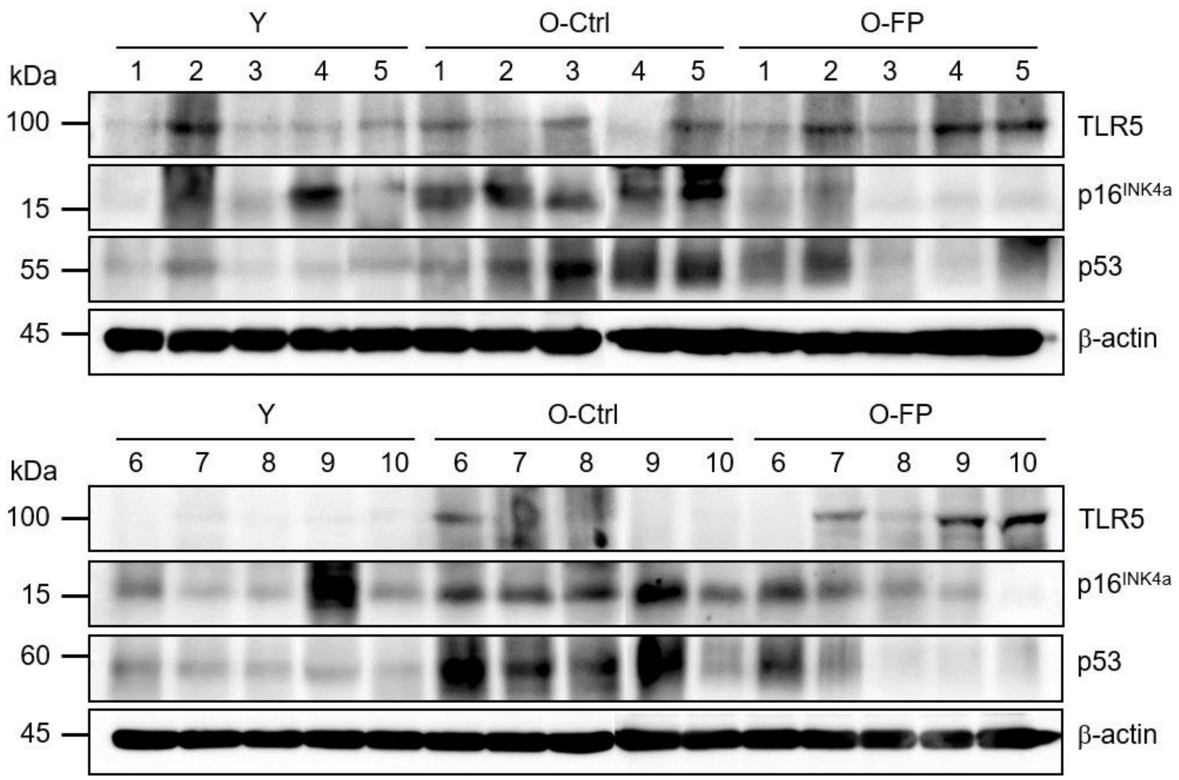

Supplementary Figure 9. Aging marker expression in intestine. After the eight times of FP or vehicle administration, intestine were isolated and analyzed the expression of aging marker proteins (n=10 biological independent repeats) by Western blotting with anti-TLR5, anti-p16^{INK4a} and anti-p53 antibodies. β-actin was used as the loading control. Source data are provided as a Source Data file.

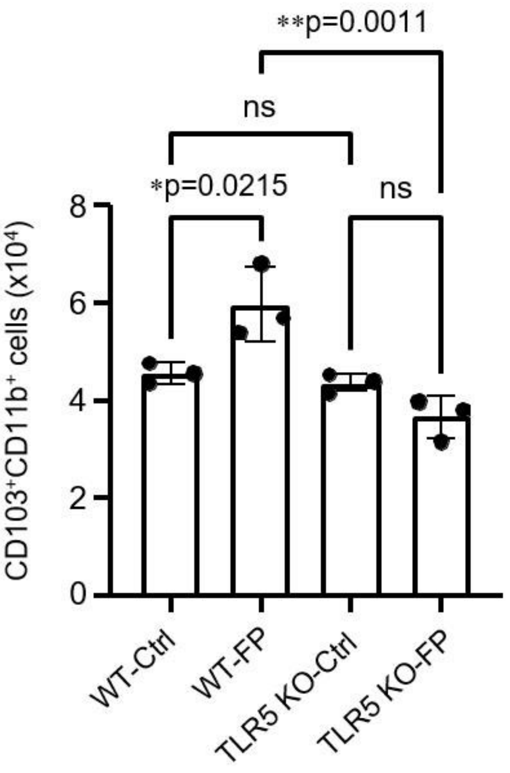

Supplementary Figure 10. Mucosal CD103^{+}cDC2 respond to FP immunization in a TLR5-dependent manner. Wild-type (WT) or TLR5 knockout (KO) mice were immunized intranasally with FP for 24 h and then the numbers of conventional dendritic cells (cDCs) were analyzed by flow cytometry in mesenteric lymph nodes (MLN). In the MLN of WT mice, intranasally immunization with FP resulted in an increase in the absolute numbers of CD103^{+}CD11b^{+} cDC2, which was abrogated in TLR5 KO mice (n=3 biological independent repeats). Error bars represent mean ± SEM. $^{*}P < 0.05$, $^{**}P < 0.01$ using the one-way ANOVA with Tukey's test for multiple comparisons. Source data are provided as a Source Data file.

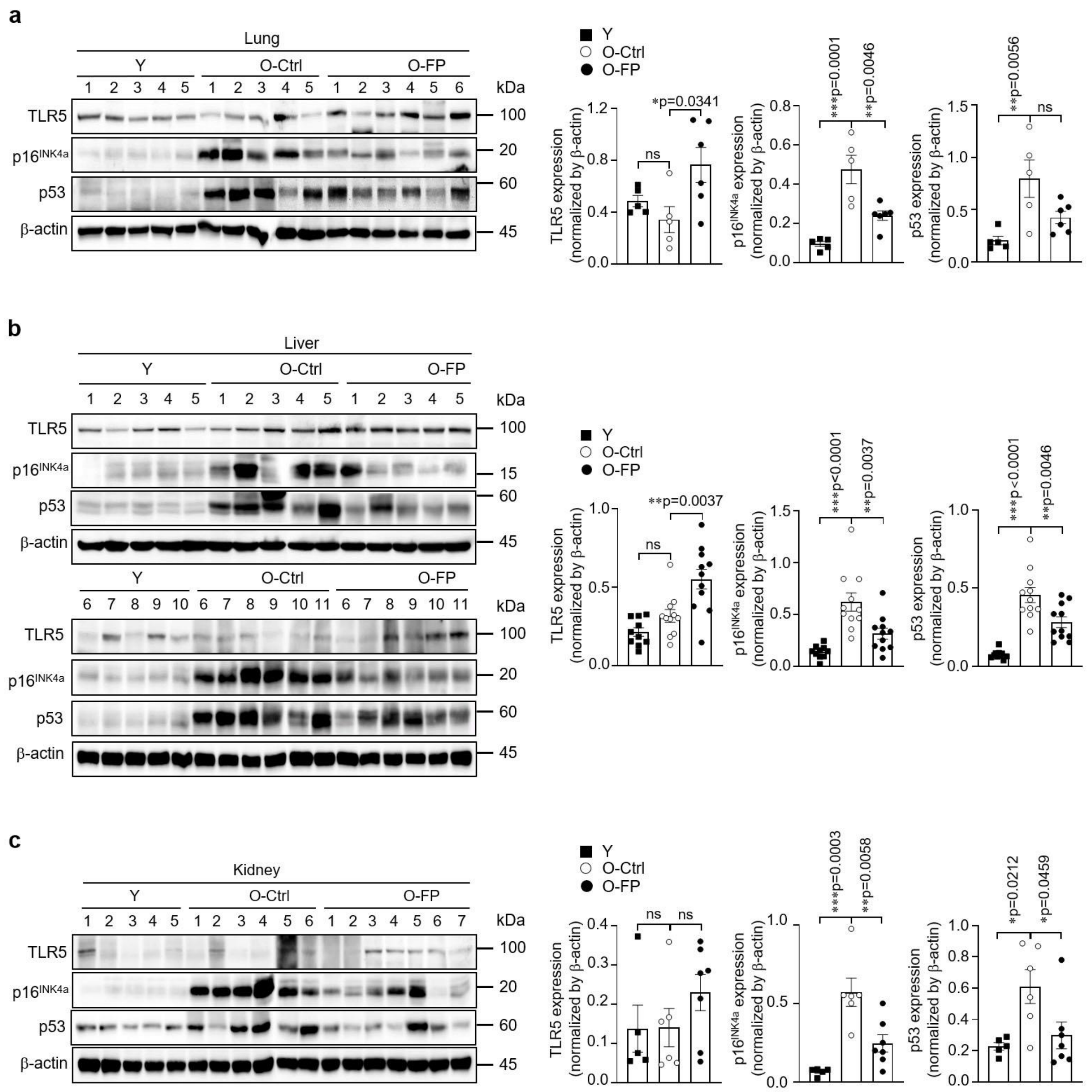

Supplementary Figure 11. Expression of TLR5 and aging markers in various tissues. After the eight times of FP or vehicle administration, the tissues were isolated and analyzed the expression of proteins in the lung (a, n=5 samples/Y; n=5 samples/O-Ctrl; n=6 samples/O-FP), liver (b, n=10 samples/Y; n=11 samples/O-Ctrl; n=11 samples/O-FP group), and kidney (c, n=5 samples/Y; n=6 samples/O-Ctrl; n=7 samples/O-FP) by Western blotting with anti-TLR5, anti-p16^{INK4a}, and anti-p53 antibodies. β-actin was used as the loading control. The data are represented by quantitative graphs. Error bars represent mean ± SEM. $^{*}P<0.05$, $^{**}P < 0.01$, $^{***}P < 0.001$ using the one-way ANOVA with Tukey's test for multiple comparisons. ns, not significant. Source data are provided as a Source Data file.

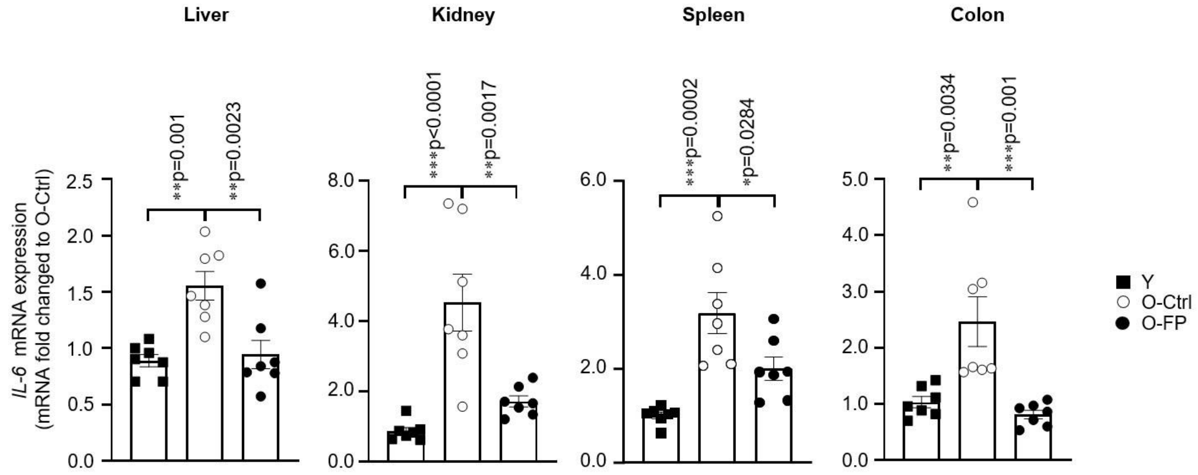

Supplementary Figure 12. IL-6 mRNA expression in various tissue. After the eight times of FP or vehicle administration, mRNA was isolated and analyzed by qPCR with IL-6 primer in the liver, kidney, spleen, and colon of mice (n=7 biological independent repeats) by qPCR. Relative RNA expression was normalized to glyceraldehyde 3-phosphate dehydrogenase (GAPDH) expression as an internal control. The data are represented by quantitative graphs. Error bars represent mean ± SEM. $^{*}P<0.05$, $^{**}P < 0.01$, $^{***}P < 0.001$ using the one-way ANOVA with Tukey's test for multiple comparisons. Source data are provided as a Source Data file.

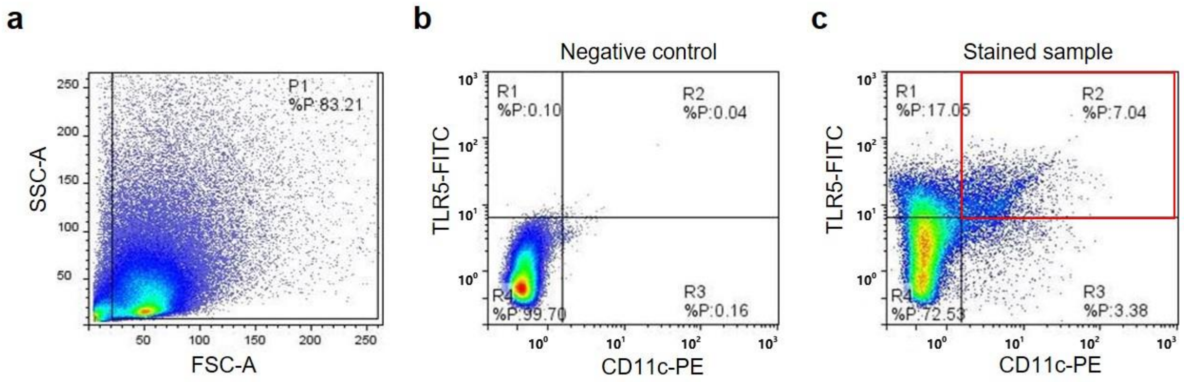

Supplementary Figure 13. Gating strategy for the analysis of CD11c and TLR5 expression in Lamina propria cells. (a) Total lamina propria (Lp) cells were gated based on size and granularity using forward scatter area (FSC-A) and side scatter area (SSC-A). (b) The untreated negative control was used to set the scale. (c) Lp cells were further analyzed upon different expression patterns of surface markers CD11c and TLR5.

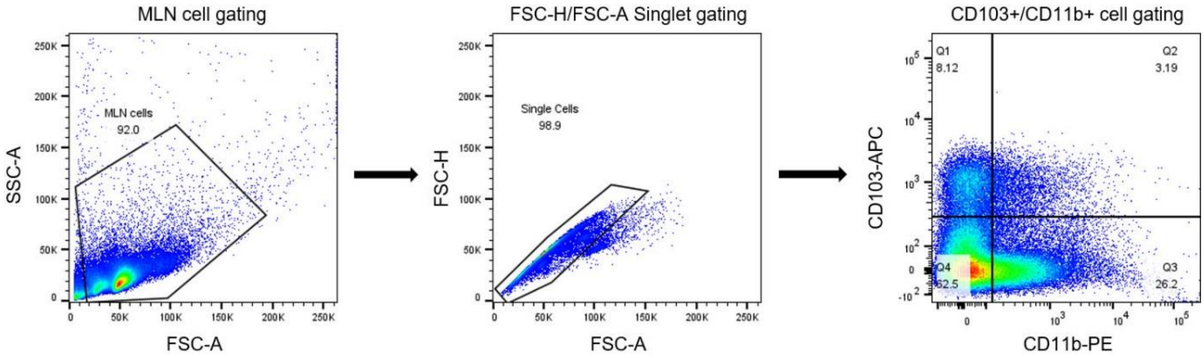

Supplementary Figure 14. Gating strategy for the analysis of conventional dendritic cells in the mesenteric lymph nodes. Total mesenteric lymph node (MLN) cells were gated based on size and granularity using forward scatter area (FSC-A) and side scatter area (SSC-A). Conventional dendritic cells (cDCs) were further analyzed upon different expression patterns of surface markers CD103 and CD11b.

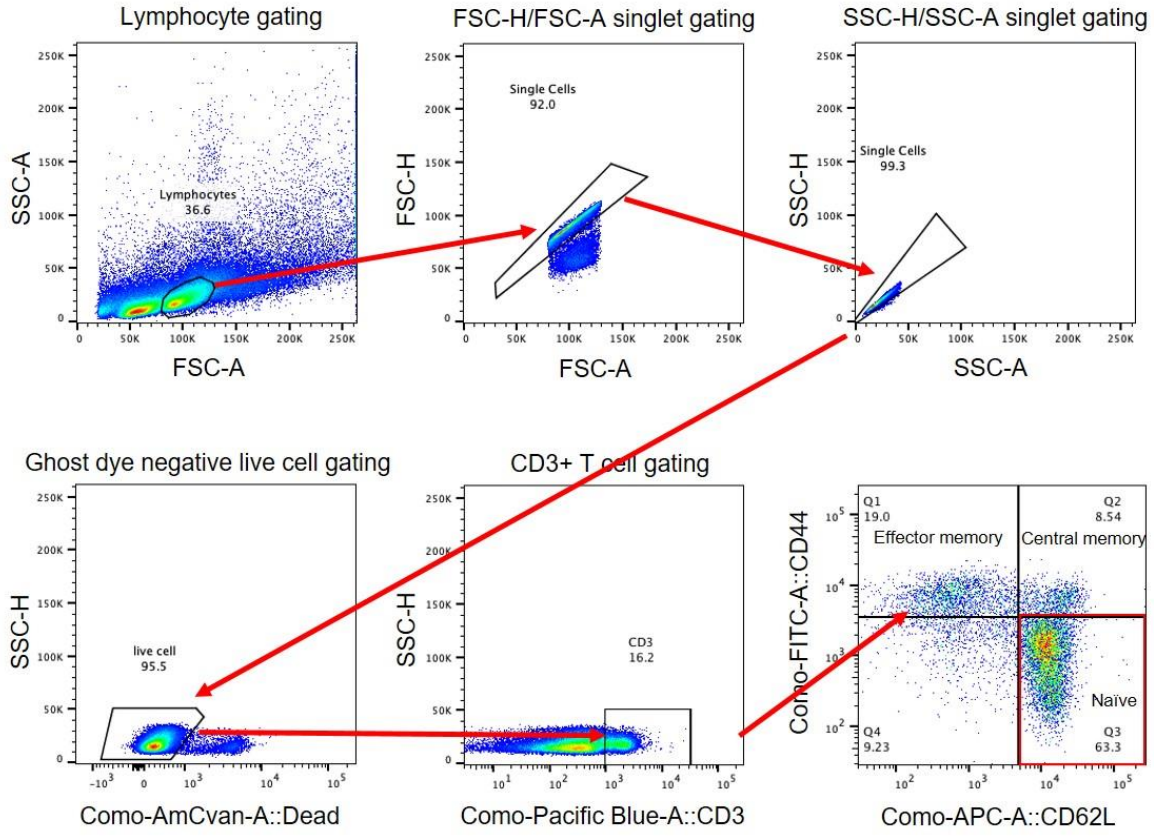

Supplementary Figure 15. Gating strategy T cell subsets from total splenocytes. Total lymphocytes were gated based on forward scatter area (FSC-A) and side scatter area (SSC-A) parameters. Singlets were firstly selected by using FSC-A and forward scatter height (FSC-H) and then discriminated by SSC-A and side scatter height (SSC-H) parameters. After gating live cells, $CD3^+$ cells were gated as the total T cell population. To classify $CD3^+$ T cell population into effector memory, central memory, and naïve subsets with CD44 and CD62L markers.

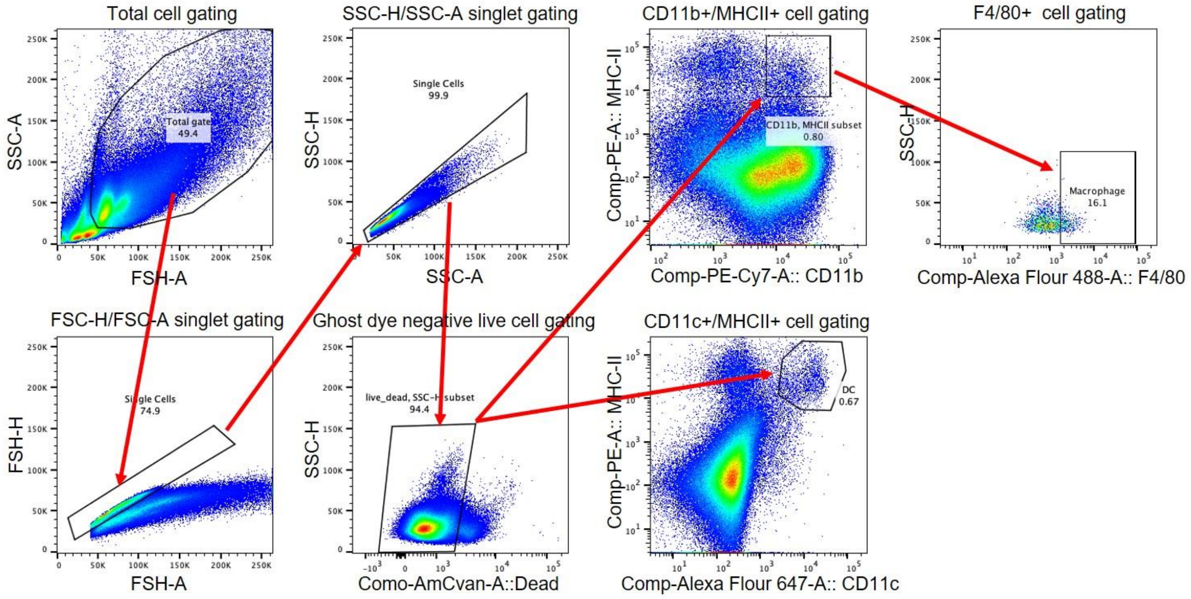

Supplementary Figure 16. Gating strategy of macrophage and dendritic cells from total splenocytes. Total splenocytes were gated based on forward scatter area (FSC-A) and side scatter area (SSC-A) parameters. Singlets were firstly selected by using FSC-A and forward scatter height (FSC-H) and then secondly discriminated by SSC-A and side scatter height (SSC-H) parameters. Within live cells, CD11b$^+$MHCII$^+$ cells were gated, and then F4/80$^+$ cells were analyzed as macrophages population. Total DCs were gated as CD11c$^+$MHCII$^+$ population.

Supplementary Table 1. Incidence of Liver abnormality

Treatment Regimens	Liver abnormality[a]
Saline	7/10 (70%)
FlaB+PspA (Mix)	4/11 (36%)
FlaB-PspA (Fusion)	1/11 (9%)**

[a] Number of mice with abnormality (tumor, cirrhosis) in liver tissues.
Visual inspection only.
Statistical significance was determined using the two-sided χ2 test.
***P=0.0041* compared with Saline administrated group.
Source data are provided as a Source Data file.

Supplementary Table 2. The FPNI-dependent extension of median and maximal survival in female and male mice.

Sex	Group	N	Mean Lifespan (days)	%Change Mean	T-test *p*-value	Median (days)	%Change Median	WA *p*-value	Maximum (days)	%Change Maximum	WA *p*-value
Female	O-Ctrl	25	860.48 ± 22.18			854			1010		
	O-FP	25	963.08 ± 28.26	+ 11.9	0.0063	946	+ 10.8	0.0465	1186	+ 17.4	0.1895
Male	O-Ctrl	26	879.15 ± 25.89			902			1051		
	O-FP	26	949.35 ± 30.12	+ 8.0	0.0416	968	+ 7.3	0.5973	1163	+ 10.7	0.1906

Note: N, number of mice; T-test, two-tailed Student's *t*-test; WA, Wang-Allison test; Maximum, 90th percentile age
FP, FlaB-PspA fusion proteins

Supplementary Table 3. Score mean of mouse phenotypes of hair status, cataract, and rectal prolapse in both of female and male.

Age of animal (Months)	Administraion frequences	Female			Male		
		Group	Hair status	Cataract	Group	Hair status	Cataract
21	0	O-Ctrl (n=46)	1.24 ± 0.07	1.02 ± 0.02	O-Ctrl (n=26)	1.12 ± 0.06	1.00 ± 0.00
		O-FP (n=46)	1.28 ± 0.08	1.04 ± 0.03	O-FP (n=26)	1.04 ± 0.04	1.00 ± 0.00
22	2nd	O-Ctrl (n=45)	1.78 ± 0.10	1.02 ± 0.02	O-Ctrl (n=24)	1.21 ± 0.08	1.04 ± 0.04
		O-FP (n=46)	1.43 ± 0.08 ∗p=0.0303	1.02 ± 0.02	O-FP (n=26)	1.04 ± 0.04 ∗p=0.0159	1.00 ± 0.00
23	4th	O-Ctrl (n=43)	2.12 ± 0.12	1.14 ± 0.09	O-Ctrl (n=22)	1.55 ± 0.10	1.14 ± 0.07
		O-FP (n=46)	1.63 ± 0.09 ∗p=0.0251	1.04 ± 0.03	O-FP (n=26)	1.19 ± 0.08∗	1.04 ± 0.04
24	6th	O-Ctrl (n=40)	2.88 ± 0.14	1.40 ± 0.11	O-Ctrl (n=20)	1.50 ± 0.10	1.35 ± 0.11
		O-FP (n=43)	1.91 ± 0.12 ∗∗∗p=0.0001	1.07 ± 0.04 ∗p=0.0456	O-FP (n=24)	1.25 ± 0.09	1.08 ± 0.06 ∗p=0.029
25	8th	O-Ctrl (n=36)	3.17 ± 0.14	1.67 ± 0.14	O-Ctrl (n=19)	1.95 ± 0.08	1.37 ± 0.11
		O-FP (n=43)	2.02 ± 0.12 ∗∗∗p<0.0001	1.12 ± 0.05 ∗∗p=0.0019	O-FP (n=21)	1.48 ± 0.10 ∗p=0.014	1.05 ± 0.05 ∗p=0.0113

Score: 1 (Good) ~ 4 (Severe)
Score of mouse phenotypes is shown as mean ± SEM.
Statistical significance was determined by using $\chi 2$ test
∗*P<0.05*, ∗∗*P<0.01*, ∗∗∗*P<0.001*, Ctrl vs. FP-administrated mice.

Supplementary Table 4. The FP- or FP MUT-dependent phenotypes of old mice.

Age of animal (Months)	Administration frequencies	Female		
		Group	Hair status	Cataract
21	0	Ctrl (n=25)	1.84 ± 0.16	1.88 ± 0.26
		FP (n=25)	1.89 ± 0.17	1.96 ± 0.23
		FP MUT (n=25)	1.88 ± 0.16	1.88 ± 0.19
25	8th	Ctrl (n=19)	3.26 ± 0.17	2.68 ± 0.22
		FP (n=23)	2.09 ± 0.22∗∗	2.00 ± 0.22
		FP MUT (n=20)	2.85 ± 0.23	2.70 ± 0.27

Score: 1 (Good) ~ 4 (Severe)
Score of mouse phenotypes is shown as mean ± SEM
Statistical significance was determined by using the $\chi 2$ test
∗∗*P=0.0035*, Ctrl vs. FP-administrated mice.

Supplementary Table 5. The FP- or FP MUT-dependent extension of median and maximal survival in female mice.

Sex	Group	N	Mean Lifespan (Day)	%Change Mean	ANOVA *p*-value	Median Lifespan	%Change Median	WA *p*-value	Maximum Lifespan	%Change Maximum	WA *p*-value
Female	Ctrl	16	840.63 ± 23.43			854			936		
	FP	16	914.31 ± 20.92	+ 8.8	0.0427	930.5	+ 9.0	0.2852	996	+ 6.4	0.0434
	FP-MUT	16	843.00 ± 22.04	+ 0.3	0.9958	854	0.0	1.0000	936	0.0	1.0000

Note: N, number of mice; ANOVA, One-way analysis of variance; WA, Wang-Allison test; Maximum, 90th percentile age
FP-MUT, site-directed mutant FP
