# Supplementary material for: Mucosal TLR5 activation controls healthspan and longevity
Source: Nat Commun. 2024 Jan 2;15:46. doi: 10.1038/s41467-023-44263-2 (PMC10761998; doi:10.1038/s41467-023-44263-2)
Supplement: Supplementary file 2 — Description of Additional Supplementary Files [file 41467_2023_44263_MOESM2_ESM.pdf]

Description of Additional Supplementary Files

Supplementary Movie 1. Active movement of FPNI-aged mice. The video is showing that motion is improved by eight times of FPNI in aged mice (25 months). Left, Ctrl (vehicle) mice; Right, FP mice. FPNI, FlaB-PspA (FP) nasal instillation.

Supplementary Movie 2. Nasal Administration Method. This video demonstrates the procedure for intranasal administration of FPNI by delivering 8 µL of phosphate-buffered saline (PBS) containing a visible indicator to one nasal passage in mice. In the study, a total volume of 16 µL of the drug was administered across both nasal cavities, with 8 µL being delivered to each nasal passage.
