# Supplementary material for: Mucosal TLR5 activation controls healthspan and longevity
Source: Nat Commun. 2024 Jan 2;15:46. doi: 10.1038/s41467-023-44263-2 (PMC10761998; doi:10.1038/s41467-023-44263-2)
Supplement: Supplementary file 5 — Reporting Summary [file 41467_2023_44263_MOESM5_ESM.pdf]

nature portfolio

Corresponding author(s): NCOMMS-20-07880-T

Last updated by author(s): Nov 22, 2023

Reporting Summary

Nature Portfolio wishes to improve the reproducibility of the work that we publish. This form provides structure for consistency and transparency in reporting. For further information on Nature Portfolio policies, see our Editorial Policies and the Editorial Policy Checklist.

Statistics

For all statistical analyses, confirm that the following items are present in the figure legend, table legend, main text, or Methods section.

n/a	Confirmed	
☐	☒	The exact sample size (n) for each experimental group/condition, given as a discrete number and unit of measurement
☐	☒	A statement on whether measurements were taken from distinct samples or whether the same sample was measured repeatedly
☐	☒	The statistical test(s) used AND whether they are one- or two-sided<br>*Only common tests should be described solely by name; describe more complex techniques in the Methods section.*
☒	☐	A description of all covariates tested
☐	☒	A description of any assumptions or corrections, such as tests of normality and adjustment for multiple comparisons
☐	☒	A full description of the statistical parameters including central tendency (e.g. means) or other basic estimates (e.g. regression coefficient) AND variation (e.g. standard deviation) or associated estimates of uncertainty (e.g. confidence intervals)
☐	☒	For null hypothesis testing, the test statistic (e.g. F, t, r) with confidence intervals, effect sizes, degrees of freedom and P value noted<br>*Give P values as exact values whenever suitable.*
☒	☐	For Bayesian analysis, information on the choice of priors and Markov chain Monte Carlo settings
☒	☐	For hierarchical and complex designs, identification of the appropriate level for tests and full reporting of outcomes
☒	☐	Estimates of effect sizes (e.g. Cohen's d, Pearson's r), indicating how they were calculated

Our web collection on statistics for biologists contains articles on many of the points above.

Software and code

Policy information about availability of computer code

Data collection	Glucose Uptake : micro-PET/CT Scanner (Inveon Siemens Medical Solutions)<br>In Vitro Angiogenesis : phase-contrast microscope (Nikon)<br>Flow Cytometry : FACSCanto II (BD Bioscience)<br>Behavior Test : Videotracking system (SMART, PanLab S.I.)<br>Histological Analysis : Aperio ImageScope instrument (Leica Biosystems Inc.)<br>PspA-specific SIgA or IgG Antibody response : Microplate reader (Molecular Devices Corp.)<br>NF-kB Luciferase Reporter Assay : Luminometer (MicroLumatPlus LB 96 V).
Data analysis	Statistical/Data analysis: Microsoft Excel (Version 2016), Graphpad Prism (Version 8.0)<br>Image quantification : Image J (Version 1.51), Image-Pro Plus (Version 6.0), ImageScope viewing software (Version 12.4)<br>Micro-CT Image : PMOD software (Version 3.310)<br>Flow analysis : FlowJo Software (Version 10.6.2).

For manuscripts utilizing custom algorithms or software that are central to the research but not yet described in published literature, software must be made available to editors and reviewers. We strongly encourage code deposition in a community repository (e.g. GitHub). See the Nature Portfolio guidelines for submitting code & software for further information.

Data

Policy information about availability of data

All manuscripts must include a data availability statement. This statement should provide the following information, where applicable:

- Accession codes, unique identifiers, or web links for publicly available datasets
- A description of any restrictions on data availability
- For clinical datasets or third party data, please ensure that the statement adheres to our policy

All relevant data are available within the article and its supplementary information/Source Data. Source data are provided with this paper.

Research involving human participants, their data, or biological material

Policy information about studies with human participants or human data. See also policy information about sex, gender (identity/presentation), and sexual orientation and race, ethnicity and racism.

Reporting on sex and gender	N/A
Reporting on race, ethnicity, or other socially relevant groupings	N/A
Population characteristics	N/A
Recruitment	N/A
Ethics oversight	N/A

Note that full information on the approval of the study protocol must also be provided in the manuscript.

Field-specific reporting

Please select the one below that is the best fit for your research. If you are not sure, read the appropriate sections before making your selection.

- [x] Life sciences
- [ ] Behavioural & social sciences
- [ ] Ecological, evolutionary & environmental sciences

For a reference copy of the document with all sections, see nature.com/documents/nr-reporting-summary-flat.pdf

Life sciences study design

All studies must disclose on these points even when the disclosure is negative.

Sample size	No sample size calculation was performed. Sample size was chosen on the experimental approach and standard practices in the field. The sample size is commonly acceptable and sufficient to determine significant differences in biological experiments (such as 10-12 independent animals for behavioral tests and 3-7 biological replicates for biochemical assays including immunoblotting, FACS, etc. The detailed information about sample size has been mentioned in the Figure legends and Source data file.
Data exclusions	During the survival experiments, the dying mice were only reflected in the results of the survival curve and excluded for subsequent experiments. In other experiments, no data were excluded from this study.
Replication	All Western blot analysis and real time qPCR were repeated at least once. All attempts at replication were successful.
Randomization	We allocated animals randomly into different groups.
Blinding	No blinding was performed in this study. Blinding was not relevant for our study because analyses were analyst independent.

Reporting for specific materials, systems and methods

We require information from authors about some types of materials, experimental systems and methods used in many studies. Here, indicate whether each material, system or method listed is relevant to your study. If you are not sure if a list item applies to your research, read the appropriate section before selecting a response.

Materials & experimental systems

n/a	Involved in the study	
	x	Antibodies
	x	Eukaryotic cell lines
x		Palaeontology and archaeology
	x	Animals and other organisms
		Clinical data
x		Dual use research of concern
x		Plants

Methods

n/a	Involved in the study	
x		ChIP-seq
	x	Flow cytometry
x		MRI-based neuroimaging

Antibodies

Antibodies used

1. Anti-TLR5 antibody: abcam, ab13876 (1:1000)
2. Anti-p16INK4a: Invitrogen/Thermo Fisher Scientific, MA5-17142 (1:1000)
3. Anti-p53(DO-1): Santa cruz, sc-126 (1:1000)
4. Anti-Beta-actin(C4) antibody: Santa cruz, sc-47778 (1:1000)
5. Anti-mouse IgG, HRP-linked antibody: Cell Signaling Technology, #7076 (1:3000)
6. Anti-gamma H2AX (phospho S139) antibody: abcam, ab26350 (1:1000)
7. CD3 Antibody (145-2C11)[PB]: BioLegend, 100334 (1:100)
8. CD44 Antibody (IM7)[FITC]: BioLegend, 103006 (1:100)
9. CD62L (L-Selection) Antibody (MEL-14)[APC]: ebioscience, 17-0621-83 (1:100)
10. TLR5 Antibody (85B152.5)[FITC]: Novus Biologicals, NBP1-97728F (1:100)
11. CD11c Antibody (Clone HL3)[PE]: BD Pharmingen, 553802 (1:100)
12. CD103 Antibody (Clone M290) [APC]: BD Pharmingen, 562772 (1:100)
13. CD11b Antibody (Clone M1/70) [PE]: BD Pharmingen, 553311 (1:100)

Validation

1. Anti-TLR5 antibody: Host: mouse; Clonality: monoclonal; Application: WB, Flow Cyt, ICC/IF, IHC-P, IHC-Fr, IP; Reactivity: mouse, human (https://www.abcam.com/tlr5-antibody-19d7592-ab13876.html)
2. Anti-pl6INK4a antibody: Host: mouse, Clonality: monoclonal, Application: WB, IHC; Reactivity: human, mouse, rat (https://www.thermofisher.com/antibody/product/p16INK4A-Antibody-clone-1E12E10-Monclonal/MA5-17142)
3. Anti-p53(DO-1) antibody: Host: mouse; Clonality: monoclonal; Application: WB, IP, IF, IHC(P), FCM; Reactivity: mouse, rat, human (https://www.scbt.com/ko/p/p53-antibody-do-1)
4. Anti-Beta-actin(C4) antibody: Host: mouse; Clonality: monoclonal; Application: WB, IP, IF, IHC(P), [LISA; Reactivity: mouse, rat, human, avian, bovine, canine, porcine, rabbit, dictyostelium discoideum and physarum polycephalum (https://www.scbt.com/p/beta-actin-antibody-c4?requestFrom=search)
5. Anti-mouse IgG, HRP-linked Antibody: Host: horse; Application: WB; Reactivity: mouse; Conjugate: HRP (https://www.cellsignal.com/products/secondary-antibodies/anti-mouse-igg-hrp-linked-antibody/7076)
6. Anti-gamma H2AX (phospho 5139) antibody: Host: mouse; Clonality: monoclonal[9F3]; Application: WB, Flow Cyt, IP, ICC/IF, IHC-P, In situ hybridization; Reactivity: mouse, rat, rabbit, chicken, guinea pig, hamster, cow, dog, human, pig, monkey (https://www.abcam.com/gamma-h2ax-phospho-s139-antibody-9f3-ab26350.html)
7. CD3 Antibody (145-2C11)[PB]: Host: Armenian Hamster; Clonality: monoclonal; Application: Flow; Reactivity: mouse; Conjugate: Pacific Blue (https://www.biolegend.com/en-us/products/pacific-blue-anti-mouse-cd3epsilon-antibody-6370)
8. CD44 Antibody (IM7)[FITC]: Host: Rat; Clonality: monoclonal; Application: Flow; Reactivity: mouse, human; Conjugate: FITC (https://www.biolegend.com/en-us/products/fitc-anti-mouse-human-cd44-antibody-314
9. CD62L(L-Selection) Antibody (MEL-14)[APC]: Host: rat; Clonality: monoclonal; Application: Flow, ICC, IF; Reactivity: mouse; Conjugate: APC (https://www.thermofisher.com/antibody/product/CD62L-L-Selection-Antibody-clone-MEL-14-Monoclonal/17-0621-83)
10. TLRS Antibody (85B152.5)[FITC]: Host: mouse; Clonality: monoclonal; Application: Flow, Flow-cs, Flow-IC, IHC(P); Reactivity: human, mouse, canine; Conjugate: FITC (https://www.novusbio.com/products/tlr5-antibody-85b1525_nbp1-97728f)
11. CD11c Antibody (Clone HL3)[PE]: Host: hamster; Application: Flow; Reactivity: mouse; Conjugate: PE (https://www.bdbiosciences.com/us/reagents/research/antibodies-buffers/immunology-reagents/anti-mouse-antibodies/cell-surface-antigens/pe-hamster-anti-mouse-cd11c-hl3/p/553802)
12. CD103 Antibody (Clone M290) [APC]: Host: rat; Application: Flow; Reactivity: mouse; Conjugate: APC (https://www.bdbiosciences.com/ko-kr/products/reagents/ flow-cytometry-reagents/research-reagents/single-color-antibodies-ruo/apc-rat-anti-mouse-cd103.562772)
13. CD11b Antibody (Clone M1/70) [PE]: Host: rat; Application: Flow; Reactivity: mouse; Conjugate: PE (https://www.bdbiosciences.com/ko-kr/products/reagents/flow-cytometry-reagents/research-reagents/single-color-antibodies-ruo/pe-rat-anti-cd11b. 553311)

Eukaryotic cell lines

Policy information about cell lines and Sex and Gender in Research

Cell line source(s)

HEK293T Human Kidney Cell Line (CRL-3216, ATCC)

Authentication	HEK293T cell line was authenticated upon purchased from ATCC (STR profile validated).
Mycoplasma contamination	HEK293T cells were tested negative for mycoplasma contamination.
Commonly misidentified lines (See ICLAC register)	NO commonly misidentified lines were used.

Animals and other research organisms

Policy information about studies involving animals; ARRIVE guidelines recommended for reporting animal research, and Sex and Gender in Research

Laboratory animals	Female and male (C57BL/6J strain) aged mice were generated in-house (by C.H.L at KRIBB, Korea) or purchased aging mice (17 months) from the Animal Facility of Aging Science in Korea Basic Science Institute (KBSI, Korea).<br>For the lung fibrosis animal experiments, Male mice (C57BL/6J strain, 8 weeks) were generated and maintained at the Institute for Research in Biomedicine (IRB, Spain).<br>For the cDC2 cell analysis, Male TLR5 knockout mice (C57BL/6J strain, 8 weeks) were purchased from The Jackson Laboratory (Bar Harbor, ME, USA).<br>All mice were housed in a specific pathogen-free animal facility, maintained on a 12 h light/12 h dark cycle, at a temperature of 22 °C and 45% humidity, with ad libitum access to food and water.
Wild animals	This study did not involve Wild animals.
Reporting on sex	Both sexes were included in the lifespan study; however, a statistically significant difference in median lifespan was observed only in the female group. Therefore, with the exception of the lifespan study, other experiments predominantly utilized female subjects.
Field-collected samples	This study did not involve samples collected from the field.
Ethics oversight	All the mouse procedures were conducted followig the guidelines of the Animal Care and Use Committee of Chonnam National University (Approval number: CNU IACUC-H-2019-23). Experimental protocols for lung fibrosis animal model were approved by the Ethical Committee for Animal Experimentation (CEEA) of the Scientific Park of Barcelona (PCB license number CEEA-18-012) and the Government of Catalunya and complied with their ethical regulations. For the lung fibrosis animal experiments, all mice were maintained at the Institute for Research in Biomedicine (IRB) under specific pathogen-free conditions following the recommendations of the Federation of European Laboratory Animal Science Associations (FELASA).

Note that full information on the approval of the study protocol must also be provided in the manuscript.

Clinical data

Policy information about clinical studies

All manuscripts should comply with the ICMJEguidelines for publication of clinical research and a completedCONSORT checklist must be included with all submissions.

Clinical trial registration	*Provide the trial registration number from ClinicalTrials.gov or an equivalent agency.*
Study protocol	*Note where the full trial protocol can be accessed OR if not available, explain why.*
Data collection	*Describe the settings and locales of data collection, noting the time periods of recruitment and data collection.*
Outcomes	*Describe how you pre-defined primary and secondary outcome measures and how you assessed these measures.*

Plants

Seed stocks	*Report on the source of all seed stocks or other plant material used. If applicable, state the seed stock centre and catalogue number. If plant specimens were collected from the field, describe the collection location, date and sampling procedures.*
Novel plant genotypes	*Describe the methods by which all novel plant genotypes were produced. This includes those generated by transgenic approaches, gene editing, chemical/radiation-based mutagenesis and hybridization. For transgenic lines, describe the transformation method, the number of independent lines analyzed and the generation upon which experiments were performed. For gene-edited lines, describe the editor used, the endogenous sequence targeted for editing, the targeting guide RNA sequence (if applicable) and how the editor was applied.*
Authentication	*Describe any authentication procedures for each seed stock used or novel genotype generated. Describe any experiments used to assess the effect of a mutation and, where applicable, how potential secondary effects (e.g. second site T-DNA insertions, mosiacism, off-target gene editing) were examined.*

Flow Cytometry

Plots

Confirm that:

- [ ] The axis labels state the marker and fluorochrome used (e.g. CD4-FITC).
- [ ] The axis scales are clearly visible. Include numbers along axes only for bottom left plot of group (a 'group' is an analysis of identical markers).
- [ ] All plots are contour plots with outliers or pseudocolor plots.
- [x] A numerical value for number of cells or percentage (with statistics) is provided.

Methodology

Sample preparation	To isolated splenocytes and thymocytes from individual mice, tissues were dissociated in RPMI 1640 medium supplemented with 10% FBS, 1% P/S, 1% L-glutamine, and 0.1% Beta-mercaptoethanol. After harvesting single-cell suspensions through Cell Strainers (60 um), the cells were washed with flow cytometry (FACS) buffer and incubated for 2 min in ACK lysing buffer, followed by centrifugation and washing with PBS, and resuspension in FACS buffer for FACS analysis.<br>To isolate lamina propria cells from individual mice, the small intestine of each mouse containing the same area was collected and weighed. Peyer's patches and fat were removed, and the tissue was flushed with cold PBS and cut into 0.5 cm pieces. Epithelium was dissociated by incubation for 40 min at 37 °C with gentle shaking in Hank's Balanced Salt Solution (HBSS) without Ca2+/Mg2+ and containing 5% FBS, 5 mM EDTA and 1 mM DTT. The remaining tissue was incubated with the components of the Lamina Propria Dissociation Kit in HBSS containing Ca2+/Mg2+ and 5% FBS for 30 min at 37 °C with gentle shaking. After incubation, the tissues was dissociated using a gentle MACS Dissociator, releasing the lamina propria cells. Then, the cells were collected after passing through a 70 m cell strainer for FACS analysis and washed in PBS supplemented with 0.5% BSA.
Instrument	BD FACSCanto II (REF 338962)
Software	FlowJo software
Cell population abundance	In this study, we quantified the number of cells and expression that were expressed only positive cells in gated cells without sorting cells.
Gating strategy	For analysis of naive T cells in splenocytes, after staining of anti-CD3-PB, CD44-FITC, and CD62L-APC on the cells, live/dead cells in the total cells were gated. Next, CD3 positive cells were gated in living cells. Then, the expression of CD44 low/ CD62L high (naive T cells) in CD3 positive cells was investigated.<br>For the analysis of CD11c and TLR5 expression in lamina propria cells: the cells were first gated based on size and granularity using FSC-A and SSC-A to eliminate debris cells. Gated cells were established for each cell type using negative control (unstained) and fluorophore positive cells. Gates were drawn to collect cells both CD11c-PE and TLR5-FITC.<br>For the analysis of cDC2 cells, the cells were first gated based on size and granularity using FSC-A and SSC-A to eliminate debris cells. Gated cells were established for each cell type using negative control (unstained) and fluorophore cells. Gates were drawn to collect cells both CD103-APC and CD11b-PE.

- [x] Tick this box to confirm that a figure exemplifying the gating strategy is provided in the Supplementary Information.
